# Estimating time-varying epidemiological parameters and underreporting of Covid-19 cases in Brazil using a mathematical model with fuzzy transitions between epidemic periods

**DOI:** 10.1371/journal.pone.0305522

**Published:** 2024-06-17

**Authors:** Hélder Seixas Lima, Unaí Tupinambás, Frederico Gadelha Guimarães

**Affiliations:** 1 Instituto Federal do Norte de Minas Gerais, Januária, MG, Brazil; 2 Graduate Program in Electrical Engineering, Universidade Federal de Minas Gerais, Belo Horizonte, MG, Brazil; 3 Department of Medical Clinic, Universidade Federal de Minas Gerais, Belo Horizonte, MG, Brazil; 4 Department of Computer Science, Universidade Federal de Minas Gerais, Belo Horizonte, MG, Brazil; Damascus University, SYRIAN ARAB REPUBLIC

## Abstract

Our study conducts a comprehensive analysis of the Covid-19 pandemic in Brazil, spanning five waves over three years. We employed a novel Susceptible-Infected-Recovered-Dead-Susceptible (SIRDS) model with a fuzzy transition between epidemic periods to estimate time-varying parameters and evaluate case underreporting. The initial basic reproduction number (*R*_0_) is identified at 2.44 (95% Confidence Interval (CI): 2.42–2.46), decreasing to 1.00 (95% CI: 0.99–1.01) during the first wave. The model estimates an underreporting factor of 12.9 (95% CI: 12.5–13.2) more infections than officially reported by Brazilian health authorities, with an increasing factor of 5.8 (95% CI: 5.2–6.4), 12.9 (95% CI: 12.5–13.3), and 16.8 (95% CI: 15.8–17.5) in 2020, 2021, and 2022 respectively. Additionally, the Infection Fatality Rate (IFR) is initially 0.88% (95% CI: 0.81%–0.94%) during the initial phase but consistently reduces across subsequent outbreaks, reaching its lowest value of 0.018% (95% CI: 0.011–0.033) in the last outbreak. Regarding the immunity period, the observed uncertainty and low sensitivity indicate that inferring this parameter is particularly challenging. Brazil successfully reduced *R*_0_ during the first wave, coinciding with decreased human mobility. Ineffective public health measures during the second wave resulted in the highest mortality rates within the studied period. We attribute lower mortality rates in 2022 to increased vaccination coverage and the lower lethality of the Omicron variant. We demonstrate the model generalization by its application to other countries. Comparative analyses with serological research further validate the accuracy of the model. In forecasting analysis, our model provides reasonable outbreak predictions. In conclusion, our study provides a nuanced understanding of the Covid-19 pandemic in Brazil, employing a novel epidemiological model. The findings contribute to the broader discourse on pandemic dynamics, underreporting, and the effectiveness of health interventions.

## 1 Introduction

**Fig 1:**
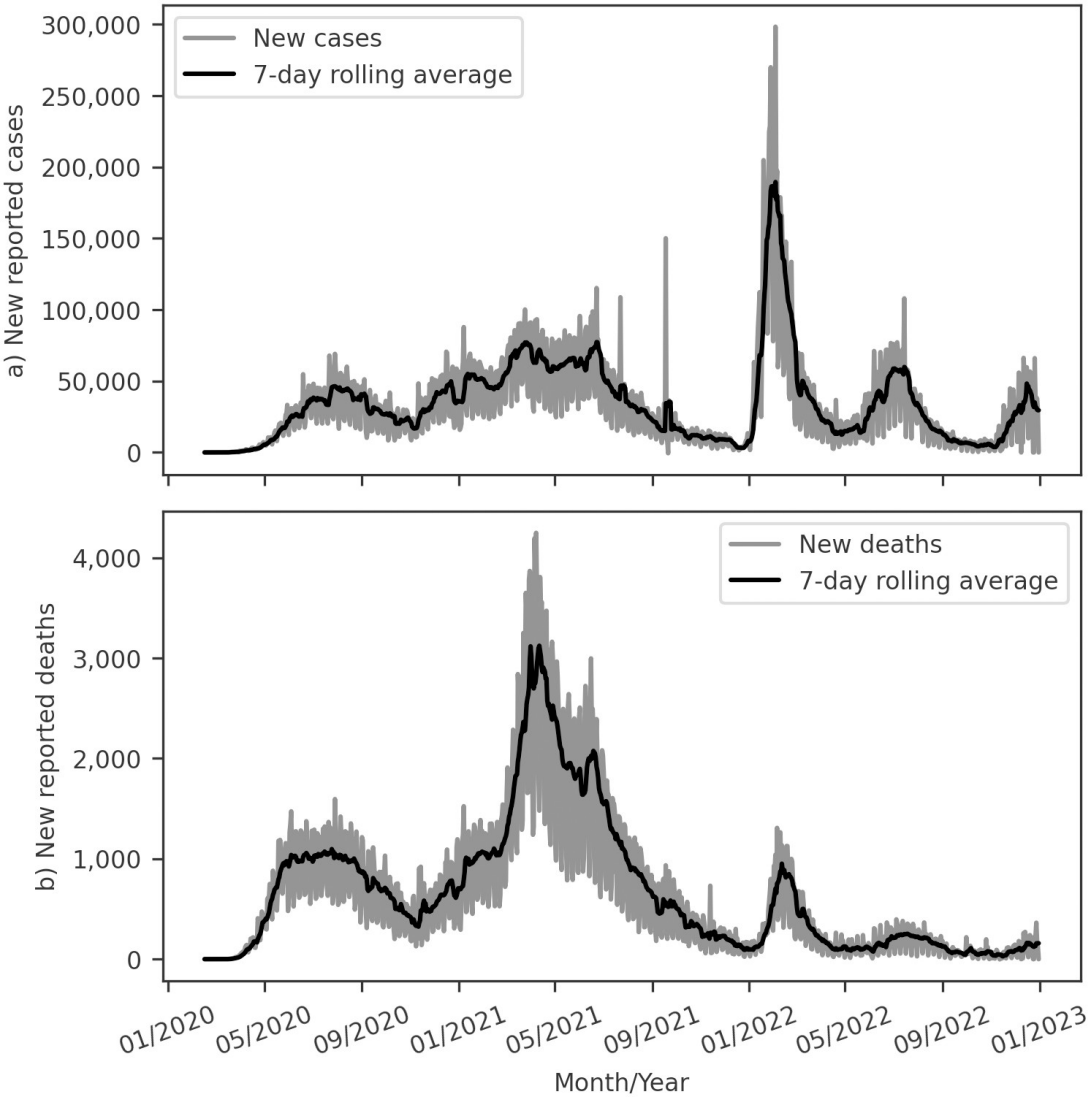
Time series of Covid-19 in Brazil at the reported date on the national monitoring panel. (a) new reported cases and (b) new reported deaths. Data from DATASUS [2].

The impact of the Covid-19 pandemic on Brazil has been profound, with the country ranking second worldwide in reported deaths [1]. By December 2022, the national health authority had reported almost 36 million cases [2] and 703 thousand deaths [3], indicating the severity of the situation. Brazil experienced five distinct waves of the pandemic over the first three years, as presented in Fig 1, with 2021 emerging as the most lethal, presenting a peak of over 3,000 deaths per day and contributing to 60% of the total deaths during the period studied [3]. Notably, 2022 marked the highest peak in reported cases, with a drastic reduction in the Case Fatality Rate (CFR) compared to earlier stages. The emergence of the Omicron variant in early 2022, characterized by high transmissibility and lower severity, also shaped the dynamics of the pandemic [4–9]. We also highlight that at the beginning of January 2022, Brazil had 78% and 67% of the population vaccinated against Covid-19 with at least one dose and fully vaccinated, respectively [4].

In this study, we aim to comprehensively reproduce the Covid-19 pandemic in Brazil over the initial three years, providing insights into the actual scale, including estimates for underreported cases. In this work, we refer to underreported cases as the number of infections estimated to have occurred in a population not captured by the surveillance system over a given time period. To achieve this, we implemented a modified Susceptible-Infected-Recovered-Dead-Susceptible (SIRDS) model, allowing us to infer changes in key epidemiological parameters: basic reproduction number (*R*_0_), Infection Fatality Rate (IFR), protected period (also named immunity period), and underreporting of cases. Our model introduces innovation by incorporating time-varying parameters through fuzzy transitions between epidemic periods. Furthermore, we explore the broader pandemic context in Brazil, examining the interplay between social isolation, vaccination coverage, and the emergence of coronavirus variants.

We conducted forecasting and sensitivity analyses to validate our model and applied it to data from countries beyond Brazil, ensuring its generalization for Spain, the United Kingdom, and the United States. During the first wave, we contextualized our findings with studies that estimated prevalence [10–16] and IFR [17–19] based in serological research. However, serological surveys have limitations in analyzing epidemics with multiple outbreaks [20], making epidemiological modeling crucial to estimating the true magnitude of prolonged epidemics. In this regard, our work relates to other studies that have presented time-varying models to reproduce changes in Covid-19 epidemiological parameters across outbreaks, considering factors like human mobility patterns, emerging variants, and vaccination [21–29]. Our work represents the first comprehensive investigation of the first three Covid-19 years in Brazil, contributing with novel insights to the existing literature. We present a comprehensive discussion of our results and a thorough review of related studies in Section 5.

Our study offers several contributions and findings, including:

- A comprehensive overview of the Covid-19 pandemic in Brazil spanning three years.
- Developing a modified SIRDS model for analyzing multi-outbreak epidemics, incorporating fuzzy transitions between epidemic periods.
- Estimation of time-varying epidemiological parameters such as *R*_0_, IFR, and immunity period.
- Generalization of our model with data from Spain, the United Kingdom, and the United States.
- Validation against robust evidence from serological surveys [10–16], demonstrating accuracy in reproducing observed trends and enhancing the reliability of our findings.
- Application of the model for forecasting outbreaks.
- Sensitivity analysis providing insights into the uncertainty of epidemiological parameters.
- Comparison of model simulations with officially reported data, revealing significant disparities and potential underreporting factors.
- Contribution to understanding the effectiveness of public health measures, such as reducing human mobility and mass vaccination, in controlling Covid-19 outbreaks. It provides evidence-based insights for formulating effective public policies and strategies to combat future outbreaks.

## 2 Background

During the early stages of the Covid-19 pandemic, the World Health Organization (WHO) had advised the adoption of non-pharmacological interventions to prevent the spread of the virus [30]. However, the federal government in Brazil failed to comply with these recommendations and neglected to coordinate a national response to the pandemic [31–35]. The scientific community heavily criticized President Jair Bolsonaro and his administration for promoting denialism [31–35], opposing containment measures [31, 32, 34], advocating for unproven Covid-19 treatments [31–35], discouraging vaccination [31], and downplaying the importance of wearing masks [31, 33].

**Fig 2:**
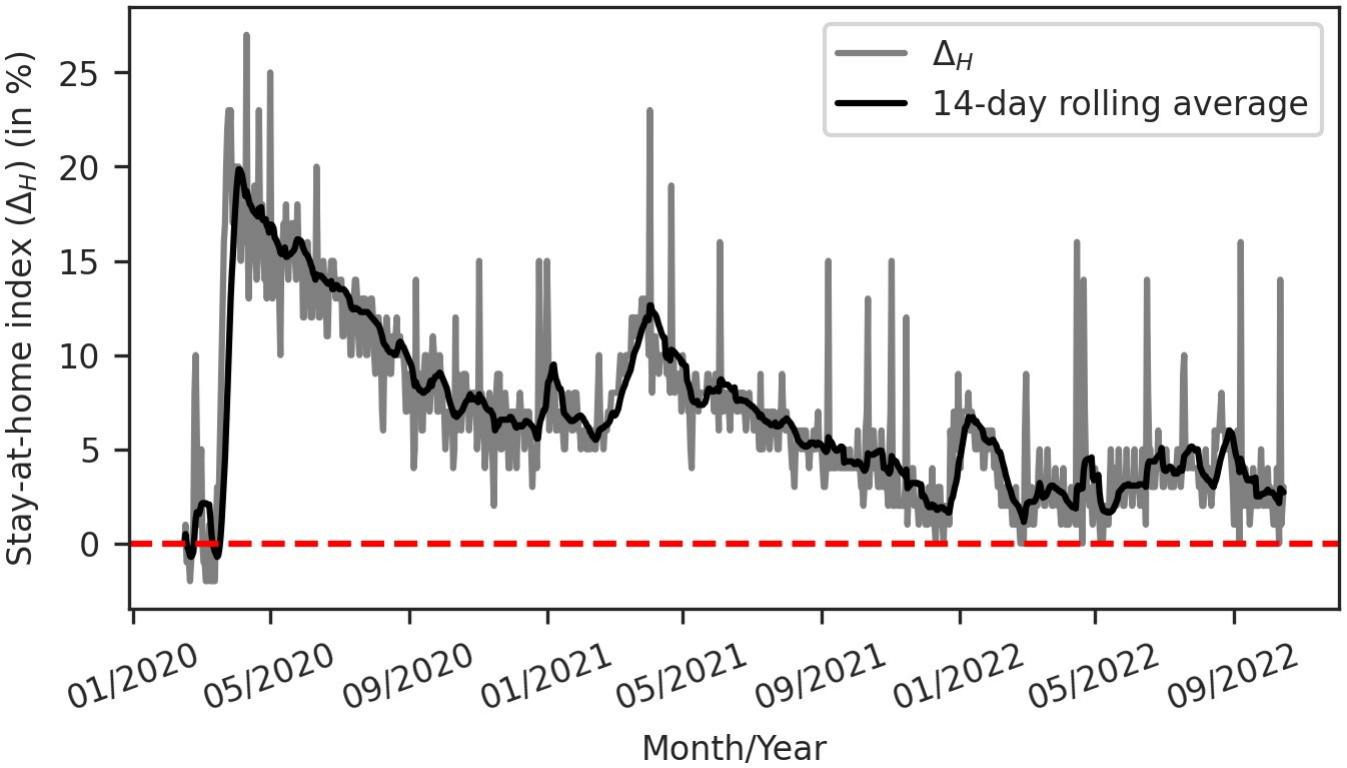
*Stay-at-home index* (Δ_*H*_) reported for the Brazilian population during the Covid-19 pandemic. The dashed horizontal line highlights the baseline (Δ_*H*_ = 0%). Data from Google Covid-19 Mobility Report [36].

Although the Brazilian federal government did not implement a national lockdown to control the spread of Covid-19, changes in the population’s mobility patterns during the pandemic were observed, either as a voluntary initiative or in response to measures taken by state and municipal governments, since the Federal Supreme Court allowed states and municipalities to take measures in favor of social isolation that oppose the federal government decision [32]. Fig 2 illustrates how the Covid-19 pandemic affected the Brazilian population’s mobility patterns. To track human mobility, we utilized data from the Covid-19 Community Mobility Report [36] produced by Google, which provided a measure of the percent change in time spent in residential places by its users in comparison to a baseline before the pandemic. We referred to this measure as the *stay-at-home index* (Δ_*H*_).

Fig 2 shows that the first notable change occurred on March 17, 2020, when the country reported its first Covid-19 death. The first outbreak in 2020 was characterized by the highest peak in Δ_*H*_, even though the death peak occurred during 2021. It is important to note that although Δ_*H*_ tends to converge to the baseline, there were periods when this trend was interrupted. For instance, during the January months in 2021 and 2022, corresponding to the school vacation period, and in mid-2021, coinciding with the peak of Covid-19 deaths, Δ_*H*_ remained high.

**Fig 3:**
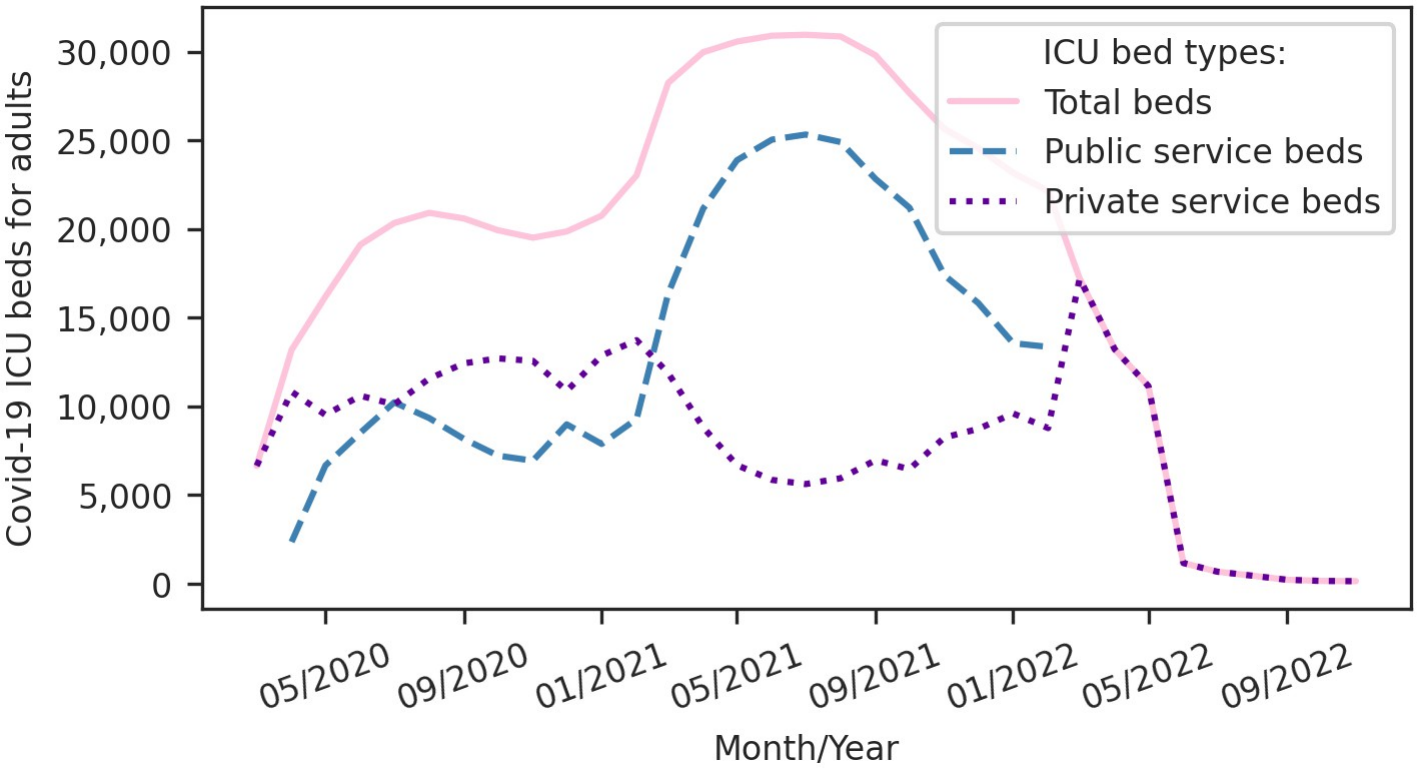
Time series of Covid-19 Intensive Care Unit (ICU) beds for adults in Brazil throughout the pandemic. Data from DATASUS [39].

The *Auxílio Emergencial* socioeconomic program was crucial in promoting social isolation and mitigating the spread of the coronavirus throughout 2020 [37, 38]. This program reached approximately 22% of the Brazilian population, paying a monthly mean of USD 154.86 from April to August 2020, continuing with reduced amounts after that period [37]. In parallel, Brazil also responded to the Covid-19 challenge by progressively increasing the number of Intensive Care Unit (ICU) beds dedicated to Covid-19 patients during the pandemic, as depicted in Fig 3 [39]. Although it does not directly impact disease transmission dynamics, this measure is a significant intervention that can potentially alleviate the IFR.

**Fig 4:**
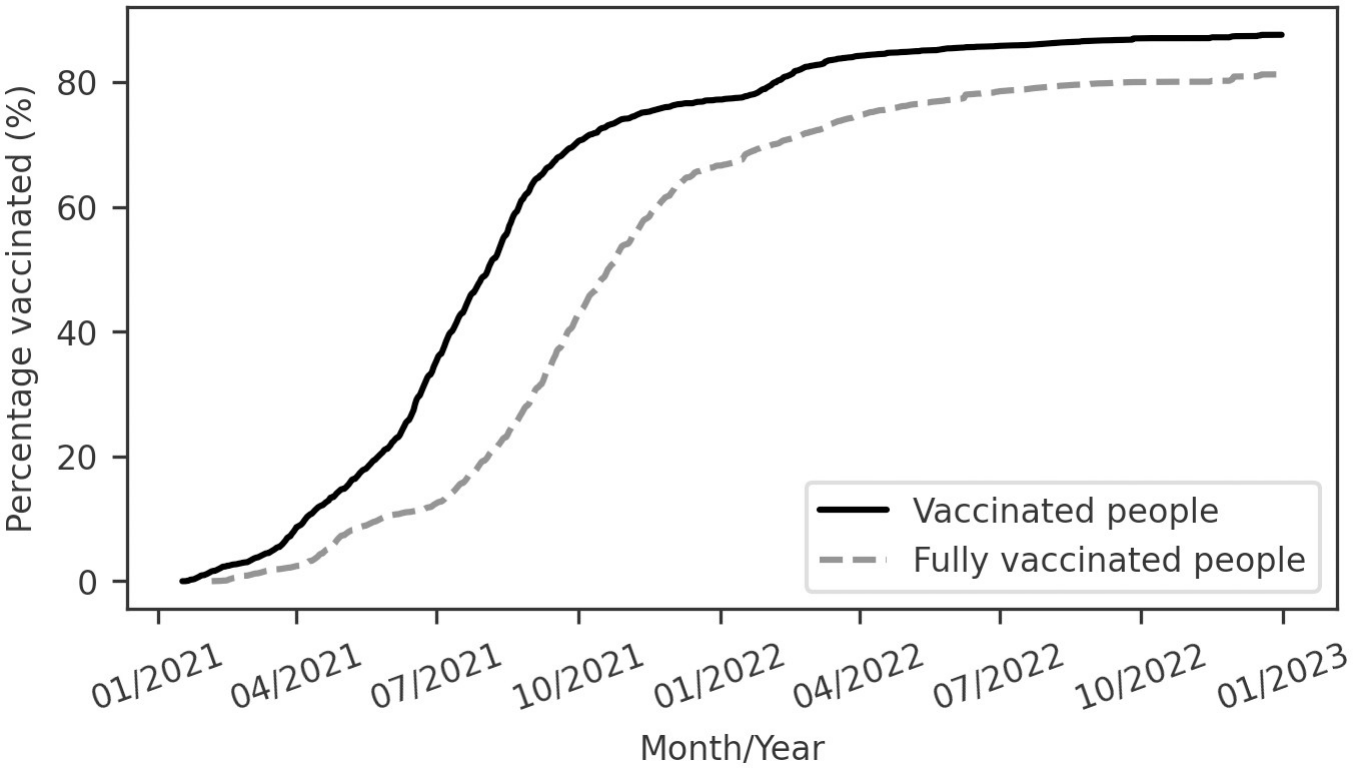
Cumulative number of individuals vaccinated and fully vaccinated against Covid-19 in Brazil. Data from Our World in Data [4].

In January 2021, Brazil initiated its Covid-19 vaccination campaign. However, only in October 2021 did half of the population receive the complete dosage required for full vaccination against Covid-19 [4], as shown in Fig 4. This situation means that during the death peak in 2021, the worst moment of the pandemic in the country, the percentage of fully vaccinated individuals was still relatively low.

**Fig 5:**
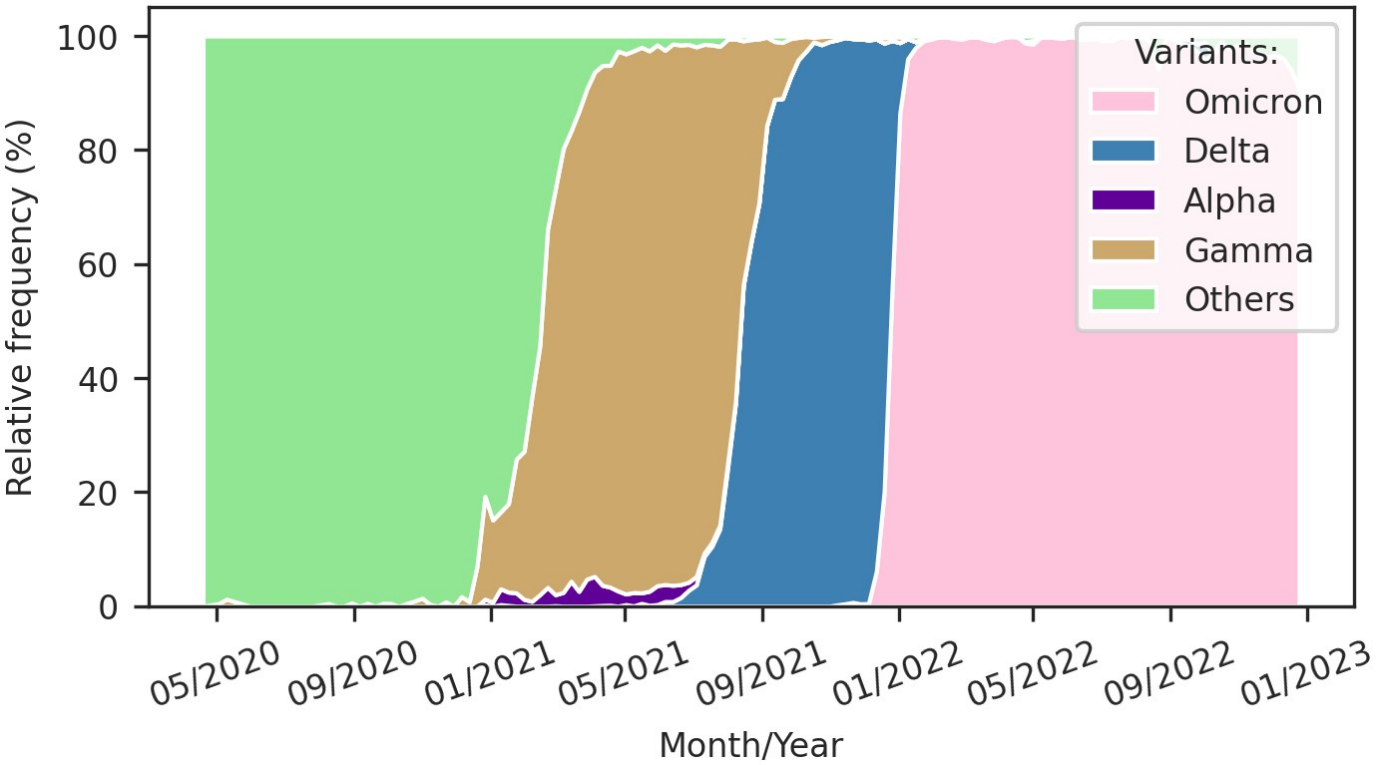
Temporal evolution of Covid-19 variants in Brazil. Relative frequency (%) of different Covid-19 variants over time, based on data from the GISAID database [40].

Another relevant topic for understanding Covid-19 in Brazil is the occurrence of coronavirus variants in the country. We analyzed the prevalent coronavirus variants using data from GISAID, a global science initiative that provides open access to genomic data of SARS-CoV-2 [40]. Fig 5 shows that the emergence of the Gamma variant coincides with the second wave of the virus in the country. This variant was initially identified in the Brazilian state of Amazonas and has been associated with increased transmissibility, higher mortality, and immune evasion, resulting in reinfections and potentially reduced efficacy of vaccines and neutralizing antibodies [41]. During the middle of the second Covid-19 wave in Brazil, the Delta variant became the dominant variant. Like the Gamma variant, the Delta variant exhibits increased transmissibility, mortality, and immune evasion [42]. Subsequently, simultaneously with the emergence of the third wave in Brazil, the Omicron variant swiftly became the predominant strain. Characterized by many mutations compared to its predecessors, Omicron exhibits elevated transmissibility and the ability to evade the immune response [6, 7]. However, works indicate a lower severity regarding disease outcomes [5–9].

## 3 Material and methods

### 3.1 Data source

The initial source of Covid-19 data in Brazil was the Monitoring Panel [2], which provided timely updates throughout the pandemic, incorporating daily information on new cases and deaths. However, it is essential to acknowledge that this data reflects the date of reporting by health authorities rather than the onset of symptoms or death. This characteristic has resulted in delayed information and time series marked by artificial weekly seasonality, as depicted in Fig 1.

**Fig 6:**
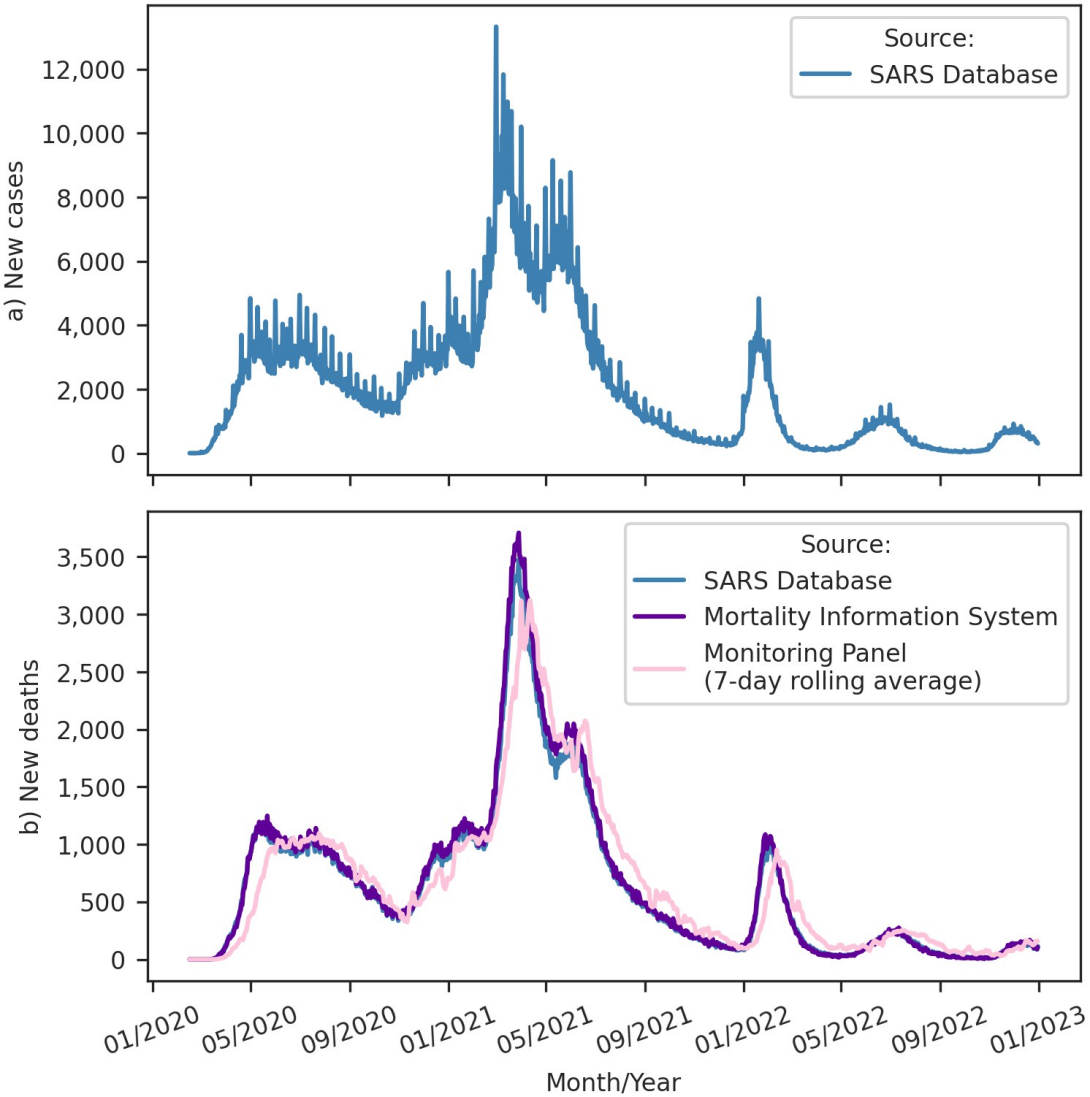
Temporal dynamics of Covid-19 in Brazil sourced from different datasets. (a) New Covid-19 cases reported by onset symptoms date of Severe Acute Respiratory Syndrome (SARS) patients, sourced from DATASUS [43]. (b) New deaths of SARS patients reported by death date [43], new Covid-19 deaths reported in Mortality Information System by death date [3], and a 7-day rolling average of new Covid-19 deaths reported in Monitoring Panel by reporting date [2], all sourced from DATASUS.

To address these limitations, we utilized the Mortality Information System [3] database from the Brazilian Health Ministry, which offers comprehensive details on all reported deaths in the country. Focusing on Covid-19 deaths, we filtered the dataset using the International Classification of Diseases (ICD) code B34.2 as the primary cause. Unlike the Monitoring Panel, this database reports the actual date of death occurrence, leading to a time series devoid of artificial seasonality, as illustrated in Fig 6b.

Furthermore, the Brazilian Health Ministry provides the Severe Acute Respiratory Syndrome (SARS) database [43], containing severe cases of Covid-19. In Fig 6b, we present Covid-19 deaths reported for SARS patients, with this dataset recording the event date and avoiding artificial seasonality in death data. Regarding severe Covid-19 cases, Fig 6a shows that only a tiny fraction of Covid-19 cases escalated in severity compared to the reported cases in Fig 1a. The SARS database reports the onset date of symptoms, a crucial contribution to analyzing disease spread dynamics. Lastly, we observe a weekly seasonality in the onset date of symptoms, although this is lower than the seasonality observed for cases reported in the Monitoring Panel [2].

Our comprehensive analysis of the Covid-19 pandemic in Brazil spans the first three years, from 2020 to 2022. Table 1 summarizes the data obtained from the investigated databases. For estimating *R*_*t*_, we utilized case data from the SARS database [43], as outlined in Section 3.2. Death data from the Mortality Information System [3] were used to fit the model proposed in this work. Additionally, case data from the Monitoring Panel [2] were employed to estimate underreporting, as detailed in Section 3.5.5. To determine the population size of Brazil, we used the 2022 Demographic Census [44] provided by the Brazilian Institute of Geography and Statistics (IBGE).

**Table 1:** Summary of Covid-19 data in Brazil (2020–2022).

Database	Data	2020	2021	2022	Total
Monitoring Panel [2]	Cases	7,675,973	14,611,548	14,043,760	36,331,281
Severe Acute Respiratory Syndrome (SARS) [43]	Cases	708,141	1,219,288	238,679	2,166,108
Monitoring Panel [2]	Deaths	194,949	424,107	74,797	693,853
Severe Acute Respiratory Syndrome (SARS) [43]	Deaths	206,468	397,498	63,952	667,918
Mortality Information System [3]	Deaths	212,704	424,461	65,392	702,557

For broader applicability, we utilized Covid-19 data from Our World in Data [4] for Spain, the United Kingdom, and the United States. This dataset was employed to illustrate the generalization of our model, as presented in Section 3.5.2. S1 Appendix provides charts with time series for these countries.

### 3.2 Estimated reproduction number

The effective reproduction number (*R*_*t*_) metric is essential for monitoring epidemics, providing insights into whether the disease is spreading or declining. Values above one indicate ongoing transmission, while values below one suggest a reduction in transmission. In our analysis of Covid-19 in Brazil, we calculated *R*_*t*_ using the time series of new cases from SARS patients [43]. This estimation involved the use of the *epyestim* library (https://github.com/lo-hfk/epyestim), a Python toolkit implementing the methodology proposed by Cori et al. [45].

To apply the *epyestim* method, we initially specified the distribution for the Covid-19 generation time, approximating it by the serial interval reported by Bi et al. [46] as ∼Gamma(2.29, 0.36), with an average of 6.36 days. Additionally, we defined the delay between infection and the onset of symptoms, representing the incubation period, as ∼Lognormal(1.57, 0.42), with an average time of 5.93 days [46].

Other parameters set in *epyestim* included a window size of 28 days to smooth the cases time series, a window size of 14 days for the final rolling average, one hundred bootstrap samples for estimating *R*_*t*_, and a prior *R*_*t*_ ∼Gamma(9.90, 9.28). The *epyestim* estimates *R*_*t*_ after cumulative cases have reached at least 12, at least one mean generation has passed since the index case, and at least one window for the final rolling average has passed since the index case.

**Fig 7:**
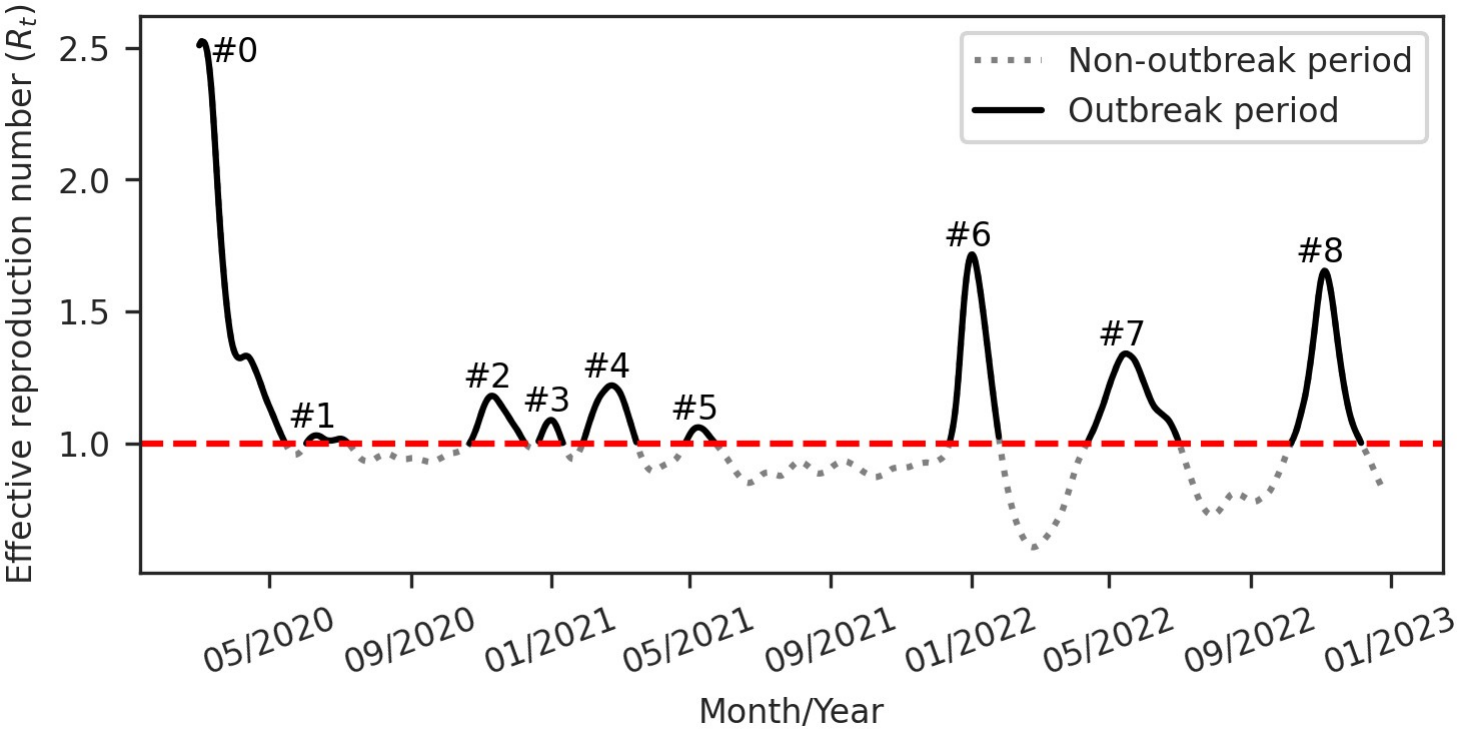
*Effective reproduction number* (*R*_*t*_) for Covid-19 in Brazil. The dashed horizontal line represents the reference value (*R*_*t*_ = 1) used to monitor epidemics. The *R*_*t*_ time series alternates between outbreak periods (solid line) and non-outbreak periods (dotted line). Nine outbreak periods, labeled from #0 to #8, were identified.

Fig 7 illustrates the time-varying *R*_*t*_ of Covid-19 in Brazil, showing that *R*_*t*_ exceeds one in different moments of the study period. The highest *R*_*t*_ values occurred in the initial pandemic phase. The period from late 2020 to mid-2021, often identified as the second wave in Brazil, is marked by recurrent *R*_*t*_ peaks, closely spaced and with lower amplitude than other periods of this pandemic. In contrast, 2022 exhibits three prominent *R*_*t*_ peaks with substantial amplitude and spaced widely in time.

We inferred the basic reproduction number (*R*_0_) by considering the *R*_*t*_ peak during the early pandemic phase. Our analysis estimated *R*_0_ for Brazil at 2.52 (95% Confidence Intervals (CI): 2.34–2.71). Our estimated *R*_0_ aligns with other studies estimating *R*_0_ for Brazil, such as *R*_0_ = 3.10 (95% CI: 2.40–5.50) [47], *R*_0_ = 2.13 (95% CI: 0.81–3.04) [48], and *R*_0_ = 2.78 (95% CI: 1.9–3.81) for a serial interval mean of 4.8 days [27]. Additionally, there are approximations, such as *R*_0_ = 3.81 (95% CI: 2.73–4.81) for a serial interval mean of 6.48 days [27].

### 3.3 Covid-19 outbreaks

Successive waves of cases and deaths commonly characterize the dynamic of the Covid-19 pandemic. Usually, we describe that the pandemic begins in Brazil with an initial wave in 2020, followed by a second wave spanning the end of 2020 through 2021, and by three distinct waves throughout 2022. However, for enhanced modeling in this study, we opt for a more fine approach by segmenting the epidemic periods based on the emergence of outbreaks. Here, we define an outbreak as when the disease spreads actively, signifying a phase when the epidemic is out of control.

To identify these outbreaks, we analyze periods where the *R*_*t*_ remains above one for a minimum duration of seven consecutive days, allowing for a maximum of seven days below this threshold during the identified outbreak period.

Consequently, our analysis identified nine Covid-19 outbreaks in Brazil over the study period, as illustrated in Fig 7. There are two outbreaks during the initial wave, four during the second wave, and three in 2022.

### 3.4 SIRDS model with fuzzy epidemic period transitions

In this study, we introduce an epidemiological model that incorporates time-varying parameters and leverages fuzzy theory to enhance the smoothness of transitions between epidemic periods. We detail the critical components of the epidemic model in Section 3.4.1. Section 3.4.2 outlines the underlying assumptions that guided our decision to propose a model with time-varying parameters and fuzzy transitions. We present the implementation of the model in Section 3.4.3. Furthermore, Section 3.4.4 delves into the presentation of the objective function and the optimization method employed in our study.

#### 3.4.1 SIRDS model core

**Fig 8:**
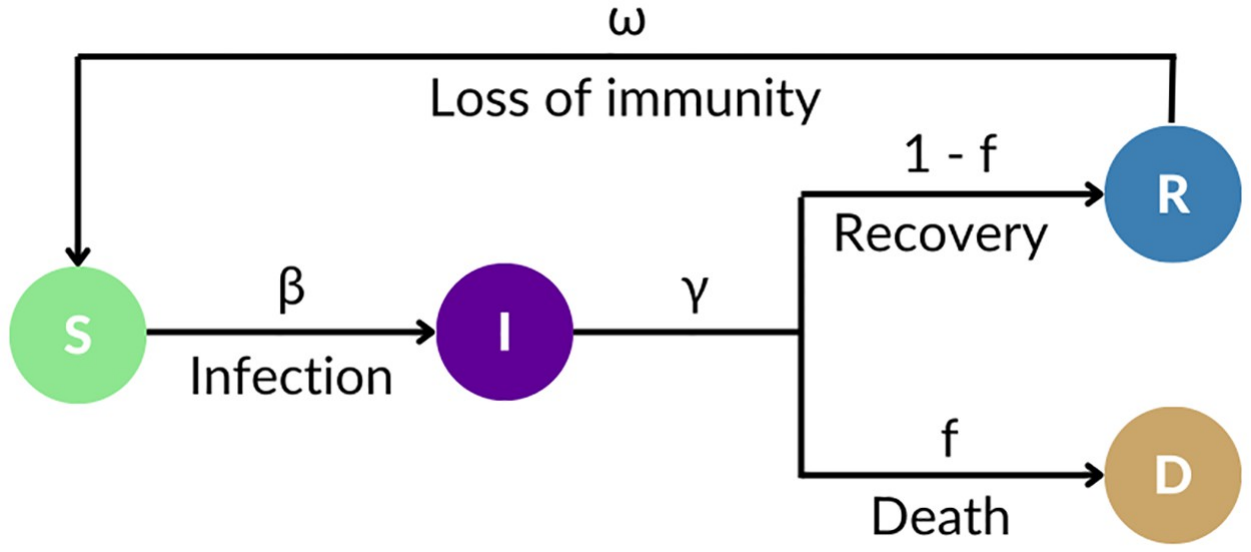
Illustration of the Susceptible-Infected-Recovered-Dead-Susceptible (SIRDS) model. Each compartment is denoted by a corresponding letter: S for Susceptible, I for Infected, R for Recovered, and D for Deceased. The model parameters include contact rate (*β*), recovery rate (*γ*), infection fatality probability (*f*), and immunity loss rate (*ω*).

The SIRDS model is commonly used in the literature to model disease epidemics where immunity after infection is temporary and a recovered individual becomes susceptible again after some time [49], as illustrated in Fig 8.

The SIRDS model, with *S* representing susceptible individuals, *I* representing infected individuals, *R* representing recovered individuals, and *D* representing deceased individuals, follows this dynamics:

- The *contact rate* (*β*) at each time unit (*t*) determines the rate at which the *I* infect the *S*.
- The *I* become either *R* or *D* at each *t* according to the *recovery rate* (*γ*) and the *infection fatality probability* (*f*).
- The *R* become *S* at each *t* due to the *immunity loss rate* (*ω*).

The *R*_0_ is a key parameter in epidemic models, representing the average number of new infections generated by each infected individual introduced into a population with no prior immunity [50]. The others SIRDS parameters *γ*, *β*, *f*, and *ω* are specified in the equations below.

$$\begin{array}{c} \gamma =\frac{1}{infectious\;period} \end{array}$$ (1)

$$\begin{array}{c} \beta =\frac{R_{0}}{infectious\;period}=\gamma R_{0} \end{array}$$ (2)

$$\begin{array}{c} f=\frac{IFR}{100} \end{array}$$ (3)

$$\begin{array}{c} \omega =\frac{1}{protected\;period} \end{array}$$ (4)

Being *N* = *S*(0) + *I*(0) + *R*(0), the compartments change over time following the four equations presented below [51, 52].

$$\begin{array}{c} \frac{dS}{dt}=-\frac{\beta IS}{N}+\omega R \end{array}$$ (5)

$$\begin{array}{c} \frac{dI}{dt}=\frac{\beta IS}{N}-\gamma I \end{array}$$ (6)

$$\begin{array}{c} \frac{dR}{dt}=\left(1-f\right)\gamma I-\omega R \end{array}$$ (7)

$$\begin{array}{c} \frac{dD}{dt}=f\gamma I \end{array}$$ (8)

#### 3.4.2 Assumptions

In this study, we propose that epidemiological parameters change during a pandemic due to modifications in mobility patterns, mutations in virus properties (such as the emergence of new variants), and fluctuations in vaccination coverage.

**Fig 9:**
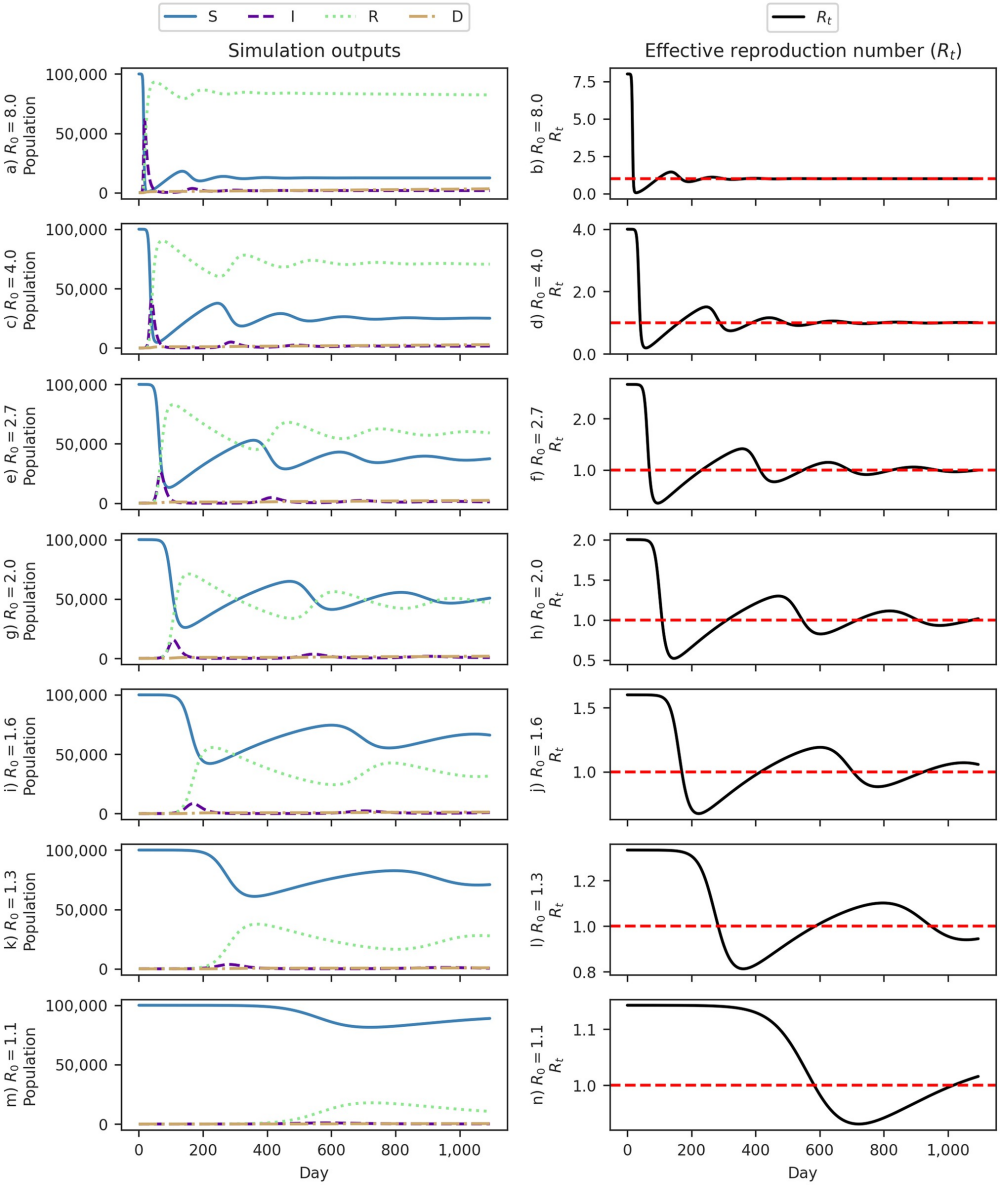
SIRDS model simulations across three years for different basic reproduction numbers (*R*_0_). Each row corresponds to a specific *R*_0_ value. The charts on the left depict simulation outputs for the Susceptible (S), Infected (I), Recovered (R), and Deceased (D) compartments. On the right side, the charts display the observed effective reproduction number (*R*_*t*_) over time, with a dashed horizontal line at the reference value (*R*_*t*_ = 1) used for epidemic monitoring.

We conducted an extensive set of 76,650 epidemic simulations spanning three years to validate these assumptions. These simulations covered a range of values for *R*_0_ (1 to 8). They included parameters such as an eight-day infectious period, a 1% IFR, a one-year protective immunity period, and an initial count of 0.14 infected individuals in a population of 100,000. Fig 9 shows the simulation results and sheds light on the dynamic patterns of *R*_*t*_.

In our empirical analysis of simulations with a static *R*_0_, we observed a pattern where each outbreak exhibited a lower *R*_*t*_ peak than its predecessor. This sequential decrease in *R*_*t*_ peaks continued until reaching a stable endemic equilibrium, as illustrated in Fig 9b and explained by Bjørnstad et al. [51].

While empirical analysis of simulations with static *R*_0_ revealed a consistent decrease in *R*_*t*_ peaks, aligning with a stable endemic equilibrium, real-world Covid-19 outbreaks displayed a different pattern. The dynamics of actual outbreaks did not conform to the consistent decrease seen in simulations, suggesting that factors like changes in mobility patterns [27, 48, 53] and the emergence of new variants [54, 55] contribute to variations in virus transmissibility, impacting the dynamic of *R*_0_.

We also observed a pattern in the initial outbreak, where *R*_*t*_ starts at the peak and then gradually declines following Eq 9 [53, 56, 57]. However, the initial outbreak coincided with a significant reduction in population mobility during the Covid-19 pandemic. Therefore, we hypothesize that the initial outbreak started with a transition pattern that changed even during the first outbreak for many locations. We conducted 18,625,950 simulations to investigate, reducing *R*_0_ by 10% to 50% at different points during the first outbreak.

$$\begin{array}{c} R_{t}=R_{0}\frac{S(t)}{N} \end{array}$$ (9)

**Fig 10:**
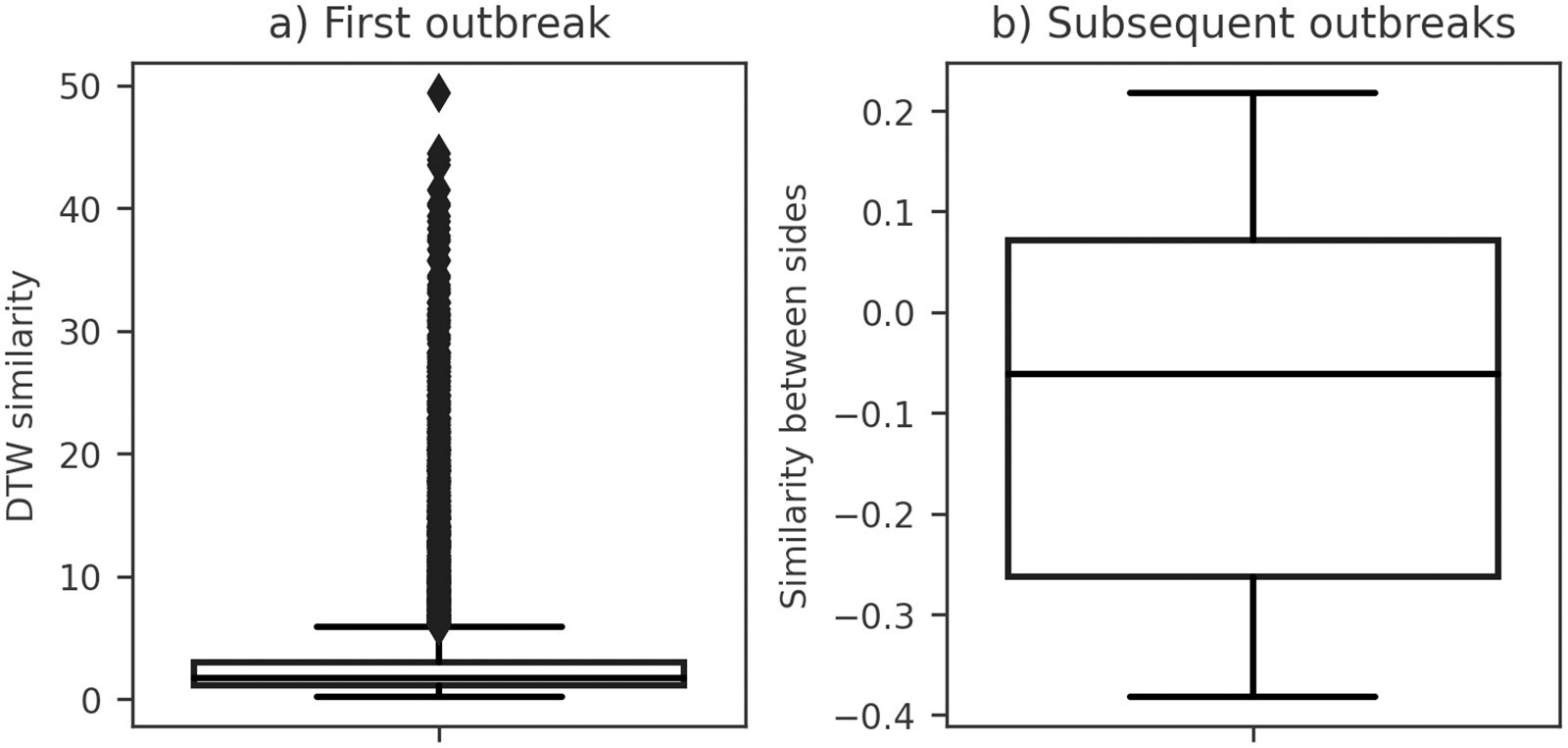
Comparative boxplots of effective reproduction number (*R*_*t*_) similarity distributions in synthetic SIRDS outbreaks. (a) Dynamic Time Warping (DTW) similarity for the first outbreak, contrasting synthetic samples with a change in basic reproduction number (*R*_0_) to their counterparts in synthetic samples without changing the *R*_0_. (b) Similarity between left and right sides for subsequent outbreaks in synthetic samples without changing the *R*_0_.

We conducted a comparative analysis of the initial outbreak simulations, comparing those with variable *R*_0_ and their counterparts in simulations with constant *R*_0_. To assess the similarities between these scenarios, we employed the FastDTW algorithm [58]. FastDTW is a heuristic algorithm with a complexity of *O*(*n*), designed as an efficient alternative to the Dynamic Time Warping (DTW) algorithm. This approach produces a distance measurement and is widely utilized for evaluating the similarity between two-time series [58]. The assessed similarities are visualized in Fig 10a, with the lowest DTW similarity recorded at 0.215.

Moving beyond the initial outbreak, we observed that the *R*_*t*_ time series of the subsequent outbreaks present a curve similar to a bell-shaped one as presented in Fig 9. To quantify the similarity between the left and right sides of these curves, we utilized the following equation:

$$\begin{array}{c} \text{similarity}=\frac{\sum_{i=p}^{e}R_{t}\left(i\right)-\sum_{i=b}^{p}R_{t}\left(i\right)}{\sum_{i=b}^{e}R_{t}\left(i\right)}, \end{array}$$ (10)

where *b* denotes the beginning point of an outbreak, *p* represents the *R*_*t*_ peak of an outbreak, and *e* signifies the end point of an outbreak. In this equation, when similarity > 0, the right side is higher than the left side; when similarity < 0, the left side is higher than the right side; and when similarity = 0, the left and right sides there is the same sum of *R*_*t*_. The assessed similarities for simulations without *R*_0_ changes are presented in Fig 10b, with the similarity between sides ranging between -0.39 and 0.22.

**Fig 11:**
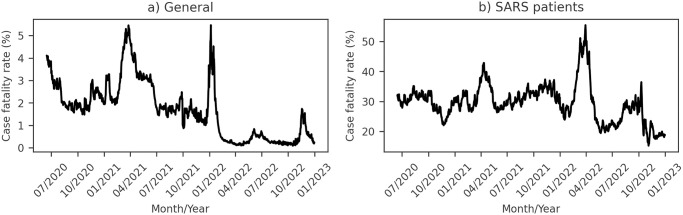
Time series of Covid-19 Case Fatality Rate (CFR) in Brazil. (a) General CFR calculated from cases reported in the Monitoring Panel [2] and deaths reported in the Mortality Information System [3]. (b) CFR calculated for Severe Acute Respiratory Syndrome (SARS) patients [43].

Additionally, we noted fluctuations in the lethality of Covid-19 across the pandemic. Fig 11a and S3 Appendix illustrate a CFR reduction from the early stages to the end of the study period for Brazil and other countries. Recognizing the impact of underreporting on this measurement, especially during pandemic peaks, we also evaluated the CFR for SARS patients in Brazil (Fig 11b). Despite the impact of underreporting, our observation suggests a gradual decrease in the lethality of Covid-19 throughout the pandemic.

Our study assumes that epidemiological parameters change during different epidemic periods. Specifically, the transition between epidemic periods for the parameter *β* tends to be rapid, while that for the parameter *f* is more gradual. Although evidence regarding the transition between epidemic periods for immunity loss is lacking, our hypothesis suggests a slow transition similar to *f*.

#### 3.4.3 Model implementation

In this section, we describe the implementation details of our SIRDS model with fuzzy epidemic period transitions, building upon the epidemiological concepts introduced in Section 3.4.1 and the assumptions outlined in Section 3.4.2.

Our model incorporates the following parameters:

- Initial infected population (*I*(0))
- Recovery rate (*γ*)
- List representing contact rates for different epidemic periods ($\vec{\beta }$)
- List representing infection fatality probabilities for different epidemic periods ($\vec{f}$)
- List representing immunity loss rates for different epidemic periods ($\vec{\omega }$)
- List denoting breakpoints for fast transition between epidemic periods ($\vec{b_{fast}}$)
- List representing transition days for smoothing fast transitions ($\vec{\tau_{fast}}$)
- List denoting breakpoints for slow transition between epidemic periods ($\vec{b_{slow}}$)

It is essential that the minimum size of the parameters $\vec{\beta }$, $\vec{f}$, and $\vec{\omega }$ be one. The parameters $\vec{b_{fast}}$ and $\vec{\tau_{fast}}$ are of the same length, both having one item less than $\vec{\beta }$. Similarly, $\vec{f}$ and $\vec{\omega }$ have the same size, both having one item more than $\vec{b_{slow}}$.

Our model instantiates a fuzzy variable to represent a fast transition (*μ*_fast_) between epidemic periods. The universe is the number of days in simulation. A fuzzy partition in this variable represents each epidemic period. These partitions are trapezoidal shapes whose peak, i.e. *μ*_fast_ = 1, extends from the correspondent breakpoint to the adjacent one. For each partition, the beginning is advanced by the correspondent transition days considering its peak beginning, and the end is delayed by the adjacent transition days considering its peak end. Thus, the total number of fuzzy partitions is $|\vec{b_{\text{fast}}}|+1$.

Our model also instantiates a fuzzy variable to represent a slow transition (*μ*_slow_) between epidemic periods. The universe also is the number of days in simulation. A fuzzy partition in this variable represents each epidemic period. These partitions are triangular shapes, beginning at the previous breakpoint, reaching the correspondent breakpoint at the top, i.e. *μ*_slow_ = 1, and ending at the next breakpoint. The exception is the last epidemic period, where there is a trapezoidal function with a shape starting at the previous breakpoint and reaching its peak from the correspondent breakpoint to the boundaries of the universe. Thus, the total number of fuzzy partitions is $|\vec{b_{\text{slow}}}|+1$.

In our model, the fuzzy variables operate with parameters *β*, *f*, and *ω* to reproduce different epidemic periods. Eq (11) defines the inference mechanism for defuzzification at day *t* for a time-varying epidemic parameter (*θ*′) using a pair of a fuzzy variable (*μ*) with *n* partitions and a list of epidemic parameters (*θ*) also with *n* items.

$$\begin{array}{c} \theta^{\prime }\left(\theta ,\mu ,t\right)=\frac{\sum_{i=0}^{n-1}\left(\mu_{i}\left(t\right)\times \theta_{i}\right)}{\sum_{i=0}^{n-1}\left(\mu_{i}\left(t\right)\right)} \end{array}$$ (11)

The algorithm for the SIRDS model with fuzzy epidemic period transitions is summarized in Algorithm 1. The simulation function takes various parameters and computes the rates of change for susceptible (*S*), infected (*I*), recovered (*R*), and deceased (*D*) populations at a given time step *t*.

**Algorithm 1** SIRDS model with fuzzy epidemic period transitions

1: **procedure** Simulation(*t*, *S*, *I*, *R*, *D*, *γ*, $\vec{\beta }$, $\vec{f}$, $\vec{\omega }$, *μ*_*fast*_, *μ*_*slow*_

2:  *N* ← *S* + *I* + *R* + *D*

3:  $\beta \leftarrow \theta^{\prime }\left(\vec{\beta },\mu_{fast},t\right)$

4:  $f\leftarrow \theta^{\prime }\left(\vec{f},\mu_{slow},t\right)$

5:  $\omega \leftarrow \theta^{\prime }\left(\vec{\omega },\mu_{slow},t\right)$

6:  *dS*/*dt* = −*βIS*/*N* + *ωR*

7:  *dI*/*dt* = *βIS*/*N* − *γI*

8:  *dR*/*dt* = (1 − *f*)*γI* − *ωR*

9:  *dD*/*dt* = *γfI*

10:  **return** *dS*/*dt*, *dI*/*dt*, *dR*/*dt*, *dD*/*dt*

This algorithm provides a comprehensive view of the model dynamics, capturing the influence of fuzzy variables on epidemic parameters and their impact on the SIRDS compartmental model.

#### 3.4.4 Parameter optimization

We employed the stochastic differential evolution algorithm [59] from the Python Scipy library for effective model parameter fitting. The objective was to minimize the discrepancies between original data and simulations for both the *death rate per 100,000 inhabitants in the 7-day moving average* (*M*) from DATASUS [3] and the *effective reproduction number* (*R*_*t*_) calculated in Section 3.2. To achieve this, we formulated a composite objective function using Eq (12):

$$\begin{array}{c} \frac{\text{MAE}(M,\hat{M})}{\bar{M}}+\frac{\text{MAE}(R_{t},\hat{R_{t}})}{\bar{R_{t}}}, \end{array}$$ (12)

where MAE denotes the mean absolute error measure, as defined by Eq (13). Here, $\hat{M}$ and $\hat{R_{t}}$ are the estimated mortality and effective reproduction numbers from our model, respectively. $\bar{M}$ and $\bar{R_{t}}$ represent the mean values of *M* and *R*_*t*_, respectively.

$$\begin{array}{c} \text{MAE}\left(y,\hat{y}\right)=\frac{1}{n}\sum_{i=1}^{n}\left|y_{i}-{\hat{y}}_{i}\right| \end{array}$$ (13)

We set the stochastic differential evolution algorithm with a maximum of 10,000 generations, a multiplier factor of five (so, the total population size is five times the quantity of model parameters), an update strategy for the best solution vector once per generation, the mutation strategy ‘best1bin’, a relative tolerance parameter of 0.01, an absolute tolerance parameter of 0 (a stringent convergence criterion), a mutation parameter range of (0.5, 1), a recombination parameter of 0.7, the Latin hypercube initialization strategy, and an additional local optimization step using the L-BFGS-B method after the global optimization process.

The input parameters for our model vary with the number of outbreaks. We first identify the outbreaks following Section 3.3. For each outbreak after the initial one, we define a breakpoint *b*_*fast*_ to represent fast transitions between epidemic periods. We estimate *R*_0_ and assess the similarity between the first outbreak *R*_*t*_ and that estimated from its corresponding *R*_0_. If the similarity is higher than 0.22, we add an adjusted *b*_*fast*_ to account for changes in disease transmissibility across the outbreak, as explained in Section 3.4.2. We also assess the similarity between sides of outbreak curves for the subsequent outbreaks. If not within the range (-0.39, 0.22), we add an adjusted *b*_*fast*_ to capture changes in disease transmissibility across the outbreak, again, as presented in Section 3.4.2. We define an initial *β* and one *β* for each *b*_*fast*_, along with one *τ* for each *b*_*fast*_.

For representing a slow transition between epidemic periods, we define one breakpoint *b*_*slow*_ for each outbreak after the initial one, considering that the interval between outbreaks is at least 180 days. Therefore, in practical terms, is *b*_*slow*_ a subset of *b*_*fast*_. We set an initial *f* and *ω*, along with one *f* and *ω* for each *b*_*slow*_.

We optimize our model considering parameter bounds presented in Table 2, a population of 100,000 individuals, and the simulation period beginning at the start of the case time series. Empirically, we observed that our model performs better when *γ* is static, so we did not optimize this parameter using the stochastic differential evolution method, as detailed in Section 3.5.1. Additionally, it is crucial to highlight that *b*_*slow*_ ⊂ *b*_*fast*_, and optimization of these parameters is unnecessary, as *b*_*fast*_ has already been optimized.

**Table 2:** Model parameter bounds for optimization.

Parameter	Description	Minimum value	Maximum value	Reference
*I*(0)	Initial quantity of infected population	$\frac{1}{\text{population}}\times 100,000$	Empirically defined	Empirical
$b_{fast}^{0}$	Adjusted breakpoint in initial outbreak for fast transition between epidemic periods	Outbreak begin	Outbreak end	Empirical
*b*_*fast*_	Breakpoint for fast transition between epidemic periods	Outbreak begin	At $R_{t}^{↑}$	Empirical
$b_{fast}^{\prime }$	Adjusted breakpoint in subsequent outbreaks for fast transition between epidemic periods	At $R_{t}^{↑}$	Outbreak end	Empirical
*τ*	Transition days for smoothing fast transitions between epidemic periods	0	56	Empirical
*β*_0_	Initial contact rate	$\gamma \bar{R_{t}}$	*γR*_0_	Empirical
$\beta_{0}^{\prime }$	Adjusted contact rate in initial outbreak	$\gamma R_{t}^{Q_{0.25}}$	$\gamma \bar{R_{t}}$	Empirical
*β*	Contact rate	$\text{max}(\text{inf}\beta_{b-1},\gamma R_{t}^{↑})$	$\gamma \bar{R_{t}}/0.3$	Empirical
*β*′	Adjusted contact rate	$\gamma R_{t}^{Q_{0.25}}$	$\gamma \bar{R_{t}}/0.3$	Empirical
*f*	IFR in probability	max(*M*, 0.0001)	min(CFR, 0.0133)	[60]
*ω*	Immunity loss rate	1/365	1/90	[61]

$R_{t}^{↑}$ : Peak of the effective reproduction number in outbreak.

$\bar{R_{t}}$ : Mean of the effective reproduction number in outbreak.

*R*_0_: Basic reproduction number.

$R_{t}^{Q_{0.25}}$ : First quartile of the effective reproduction number in outbreak.

*γ*: Recovery rate.

inf *β*_*b*−1_: Minimum bound of the previous contact rate.

IFR: Infection Fatality Rate.

CFR: Case Fatality Rate in the epidemic period.

M: Death rate per 100,000 inhabitants in the epidemic period.

### 3.5 Experiments

#### 3.5.1 Fitting the recovery period

By the insights from Voinsky et al. [62], indicating a Covid-19 infectious period spanning from 8 to 20 days, we systematically assessed these infectious periods within our model. To estimate the recovery rate (*γ*), we considered a range of values: $\gamma \in \{\frac{1}{8},\frac{1}{9},\frac{1}{10},...,\frac{1}{20}\}$. We conducted 20 model executions for each *γ* using Brazilian data. The maximum bound for the parameter initial quantity of infected population (*I*(0)) was set as the sum of case rates per 100,000 inhabitants until the 14^*th*^ day in the first outbreak (i.e., 0.043).

**Fig 12:**
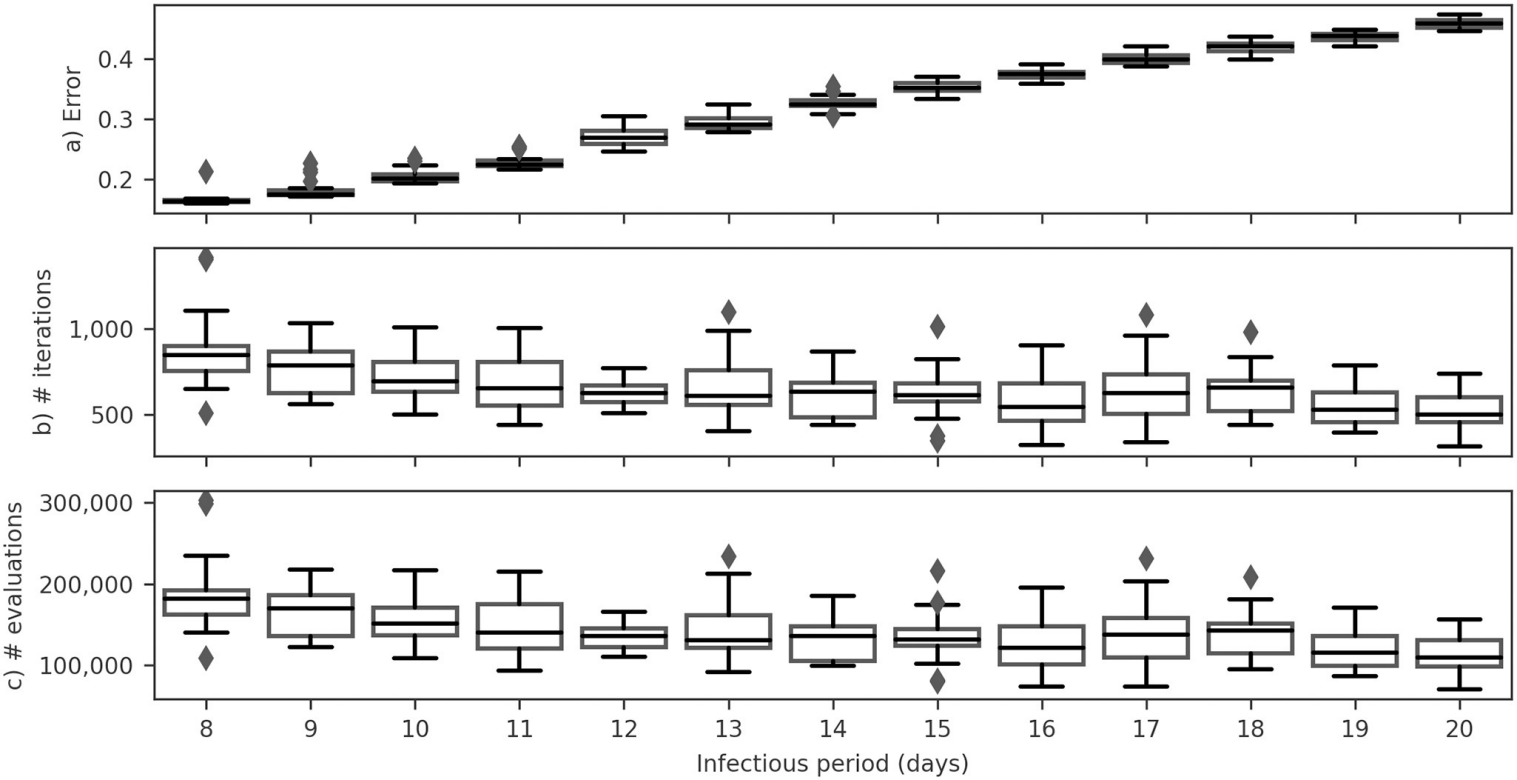
Boxplots illustrating key metrics of the model optimization process for infection periods ranging from 8 to 20 days. The boxplots depict: (a) the error in the objective function, (b) the number of iterations performed by the optimization algorithm, and (c) the number of objective function evaluations conducted by the optimization algorithm. In each boxplot, the lower and upper bounds represent the first and third quartiles, respectively. The horizontal line within the box indicates the median, while the whiskers extend to the minimum and maximum values within 1.5 times the interquartile range.

The outcomes, illustrated in Fig 12a, underscore the optimal performance achieved by our proposed model when the *infectious period* is configured to eight days, corresponding to *γ* = 1/8, which we observed error of 0.165 (95% CI: 0.162–0.170). Furthermore, Fig 12b and 12c suggest that adopting an infectious period of 8 days incurs a slightly higher optimization cost. Importantly, this incremental cost is well-justified, contributing to refining and enhancing the model outcome. Therefore, all subsequent experiments in this study were conducted with *γ* = 1/8.

In Section 4, we succinctly present the outcomes for the optimal infection period. We evaluate the effectiveness of our model using two key metrics: the Sum of Squared Errors (SSE) and the coefficient of determination (R²), defined by Eqs (14) and (15), respectively.

$$\begin{array}{c} \text{SSE}=\sum_{i=1}^{n}{\left(y_{i}-{\hat{y}}_{i}\right)}^{2} \end{array}$$ (14)

$$\begin{array}{c} R^{2}=1-\frac{\sum_{i=1}^{n}{\left(y_{i}-{\hat{y}}_{i}\right)}^{2}}{\sum_{i=1}^{n}{\left(y_{i}-\bar{y}\right)}^{2}} \end{array}$$ (15)

#### 3.5.2 Model application to other countries

To apply our model to data from Spain, the United Kingdom, and the United States, we first estimated the *R*_*t*_ time series using the methodology outlined in Section 3.2. However, adjustments were necessary for the input data of these countries. Specifically, we estimated the onset of symptoms from the new death time series, considering a delay of 19 days between onset and death [60]. The S2 Appendix includes charts displaying *R*_*t*_ time series for these countries.

We conducted 20 pandemic simulations for each country, assuming an infectious period of eight days (*γ* = 1/8). In the cases of Spain and the United States, we set the maximum bound for the parameter *I*(0) as the sum of case rates per 100,000 inhabitants until the beginning of the first outbreak time series, resulting in 0.0084 and 0.0068, respectively. On the other hand, we set the maximum bound for the United Kingdom for the parameter *I*(0) to the same value as the minimum bound for this parameter, as presented in Table 2, and set the simulation period began at the peak date of *R*_*t*_ within the initial outbreak.

#### 3.5.3 Forecasting outbreaks using the model

We utilized our model to project the dynamics of Covid-19 outbreaks in Brazil. We conducted 20 simulations for each outbreak, fitting the model from the early pandemic moment until the 21^*st*^ day and forecasting the following three months. We executed all simulations assuming an eight-day infectious period, and the remaining parameters were those employed in fitting the recovery period, as detailed in Section 3.5.1.

We compared the simulation outcomes with actual data by analyzing the objective function, Eq (12), for both fitting and forecasting samples. Also, we understand that the main contribution of a medium-term epidemic prediction is to provide insight into the total of deaths. So, we evaluated the Percentual Absolute Error (PAE), Eq (16), for the death prediction.

$$\begin{array}{c} \text{PAE}=|\frac{\sum_{i=1}^{n}y_{i}-\sum_{i=1}^{n}{\hat{y}}_{i}}{\sum_{i=1}^{n}y_{i}}|\times 100 \end{array}$$ (16)

To showcase the benefits of extending the model fitting period further into within an outbreak, we experimented with fitting the model until the 28^*th*^, 35^*th*^, 42^*th*^, 49^*th*^, 56^*th*^, 63^*rd*^, and 70^*th*^ days for the initial outbreak.

#### 3.5.4 Parameter sensitivity assessment

To evaluate the local sensitivity of our model parameters, we employed the One-Parameter-at-a-Time (OAT) method [63] using the simulations for Brazil data. The mean values estimated for the parameters in Section 3.5.1, corresponding to an infectious period of eight days (*γ* = 1/8), served as the baseline. For each parameter, we perturbed its value by 1%, 10%, and 50%, keeping other parameters fixed.

As defined in Eq (17), the absolute elasticity measurement was utilized to assess parameter sensitivity, considering the objective function as the output. An absolute elasticity greater than one indicates that the parameter is elastic, meaning higher sensitivity. In other words, a change in the parameter leads to a proportionally more significant change in the output. Conversely, an absolute elasticity of less than one suggests that the parameter is not elastic, indicating lower sensitivity, as a change in the parameter results in a proportionally smaller change in the output.

$$\begin{array}{c} \text{absolute}\;\text{elasticity}=\left|\frac{\%\;\text{change}\;\text{in}\;\text{output}}{\%\;\text{change}\;\text{in}\;\text{input}}\right| \end{array}$$ (17)

#### 3.5.5 Estimating epidemiological measurements

This section introduces measurements crucial for assessing the scale of a pandemic, as employed in this study. The Case Fatality Rate (CFR), defined by Eq (18), serves as a percentual lethality metric based on epidemiological data officially reported by health authorities. In contrast, the Infection Fatality Rate (IFR), expressed by Eq (19), serves as a percentual lethality metric derived from model-based estimations.

$$\begin{array}{c} \text{CFR}=\frac{\text{reported}\;\text{deaths}}{\text{reported}\;\text{cases}}*100 \end{array}$$ (18)

$$\begin{array}{c} \text{IFR}=\frac{\text{estimated}\;\text{deaths}}{\text{estimated}\;\text{infections}}*100 \end{array}$$ (19)

The underreporting factor, as estimated using Eq (20), represents the ratio between estimated infections by a model and reported cases by health authorities. It is essential to clarify that estimated infections encompass not only the reported cases but also the unreported cases and potentially any cases overreported by the health authorities.

$$\begin{array}{c} \text{underreporting}\;\text{factor}=\frac{\text{estimated}\;\text{infections}}{\text{reported}\;\text{cases}} \end{array}$$ (20)

## 4 Results

### 4.1 Retrospective analysis

**Fig 13:**
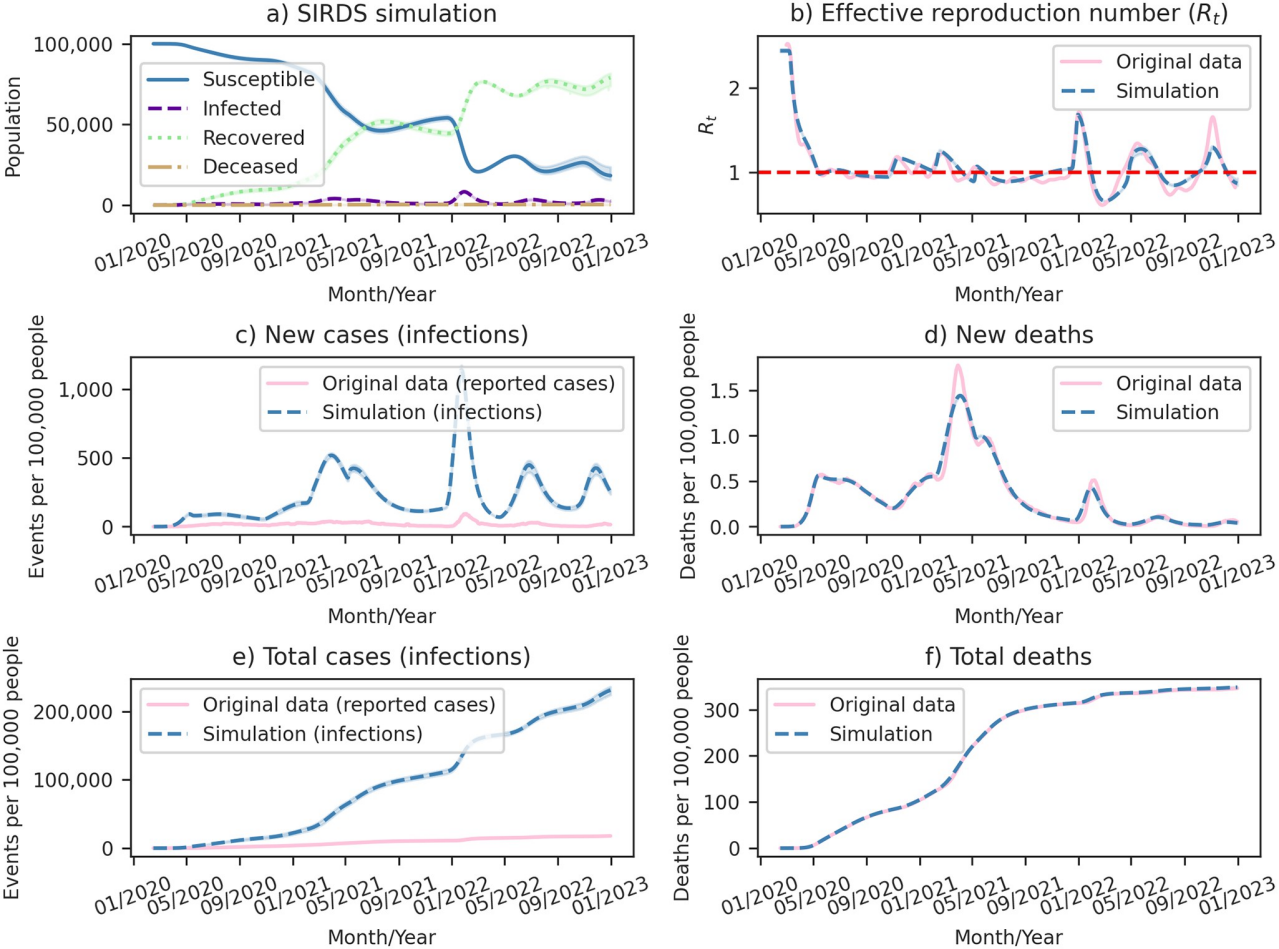
Comprehensive analysis of simulation results for Covid-19 in Brazil. (a) Model outcomes for an eight-day recovery period detailing the population compartments: Susceptible, Infected, Recovered, and Deceased. (b) Time series comparison between the effective reproduction number (*R*_*t*_) estimated directly from reported Severe Acute Respiratory Syndrome (SARS) cases and *R*_*t*_ calculated by model simulations. (c) Time series comparison between new cases reported by health authorities and new infections in model simulations. (d) Time series comparison between new deaths reported by health authorities and new deaths in model simulations. (e) Time series comparison between cumulative cases reported by health authorities and cumulative infections in model simulations. (f) Time series comparison between cumulative deaths reported by health authorities and cumulative deaths in model simulations. Shaded regions depict the 95% Confidence Interval (CI).

In this retrospective analysis, we focus on the results obtained using our model with an eight-day recovery period (*γ* = 1/8) applied to Covid-19 data from Brazil. Fig 13 provides a comprehensive overview of our model outcomes, including comparisons with the original data for various aspects such as the *R*_*t*_, new cases, new deaths, total cases, and total deaths.

Table 3 presents the quantitative evaluation of the model performance, demonstrating a low SSE and high R² for both *R*_*t*_ and new deaths, indicating a well-fitted model.

**Table 3:** Results for Covid-19 simulation with data from Brazil, Spain, United Kingdom, and United States.

Country	Error	SSE		R^2^	
		*R*_*t*_	New death rate	*R*_*t*_	New death rate
Brazil	0.165 (0.162–0.170)	0.009 (0.008–0.011)	0.004 (0.004–0.004)	0.872 (0.844–0.888)	0.967 (0.965–0.969)
Spain	0.256 (0.251–0.263)	0.054 (0.044–0.072)	0.004 (0.004–0.004)	0.786 (0.716–0.826)	0.951 (0.949–0.953)
United Kingdom	0.260 (0.244–0.274)	0.044 (0.042–0.045)	0.009 (0.007–0.011)	0.711 (0.699–0.722)	0.943 (0.926–0.957)
United States	0.126 (0.123–0.130)	0.004 (0.004–0.004)	0.001 (0.001–0.002)	0.963 (0.961–0.965)	0.971 (0.970–0.972)

Note: values are presented as the mean with 95% Confidence Interval bounds in parenthesis.

Error: the objective function error, as defined by Eq (12).

SSE: Sum of Squared Error.

R^2^: coefficient of determination.

*R*_*t*_: effective reproduction number.

New death rate: per 100,000 inhabitants.

**Fig 14:**
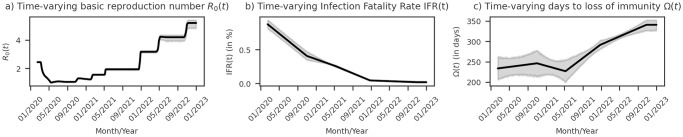
Time-varying model parameters fitted for Covid-19 in Brazil. (a) Basic reproduction number (*R*_0_) varying with time (*t*). (b) Infection Fatality Rate (IFR) varying with *t*. (c) Days to loss of immunity (*Ω*) varying with *t*. Shaded regions depict the 95% Confidence Interval (CI).

Fig 14a presents the model estimation of the *R*_0_ for Brazil, indicating an initial value of 2.44 (95% CI: 2.42–2.46). Notably, the model depicts a subsequent decrease in *R*_0_ to 1.00 (95% CI: 0.99–1.01) on May 18, 2020. Following this initial decline, the model suggests a successive increase in *R*_0_ with each outbreak, culminating in its peak value of 5.20 (95% CI: 4.83–5.41) observed in the last outbreak.

Regarding the IFR, Fig 14b suggests an initial Covid-19 lethality in Brazil of 0.88% (95% CI: 0.81%–0.94%). The model further indicates a continuous reduction in IFR throughout the outbreaks, reaching its lowest value of 0.018% (95% CI: 0.011–0.033) in the last outbreak.

The parameter about the number of days to loss of immunity, Fig 14c, presented the highest uncertainty in our model. The initial immunity period was estimated to be 234 days (95% CI: 206–262), exhibiting an increasing trend, albeit with a reduction observed in mid-2021. The last studied outbreak revealed an immunity period of 341 days (95% CI: 327–352).

Our model also estimates that 63 people (95% CI: 58–68) were infected in Brazil when the health authorities reported the first Covid-19 case. For further insights, the S4 Appendix details the fuzzy variables used in this work to facilitate smooth transitions between epidemic periods.

In conclusion, our model reveals a substantial disparity between reported cases in Monitoring Panel [2] and simulated infections, estimating a factor of 12.9 (95% CI: 12.5–13.2) more infections than the officially notified cases by Brazilian health authorities until the end of 2022. When analyzing the data for each year, we found that the simulated infections were 5.8 (95% CI: 5.2–6.4), 12.9 (95% CI: 12.5–13.3), and 16.8 (95% CI: 15.8–17.5) times higher than the reported cases in 2020, 2021, and 2022 respectively.

### 4.2 Model generalization

Table 3 illustrates the broad applicability of our model across other countries. We observe reduced errors and well-fitted coefficients of determination for both reproduction number and new deaths. Notably, the simulations for the United States exhibit the highest level of fitting. Overall, the simulations align more closely with the rate of new deaths than the reproduction number. An overview of the simulations for Spain, the United Kingdom, and the United States is available in S6 Appendix.

### 4.3 Comparisons with serological research

**Fig 15:**
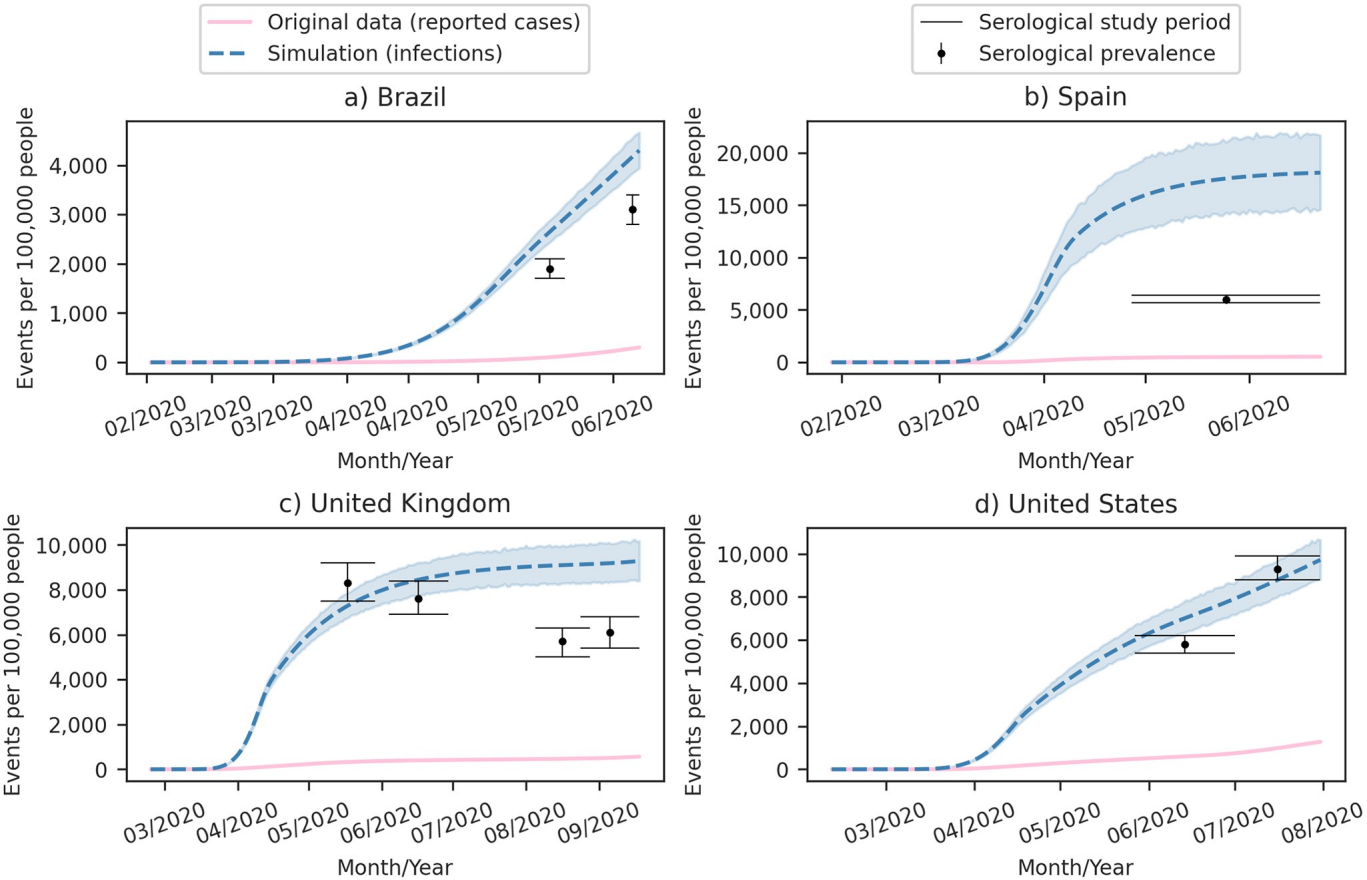
Comparison of cumulative Covid-19 infections simulated by our model, cumulative reported cases by health authorities, and serological prevalence during the early stages of the pandemic in various countries. (a) Brazil: reported cases from DATASUS [2] and serological prevalence from Hallal et al. [10]. (b) Spain: reported cases from Our World in Data [4] and serological prevalence from Perez-Gómez et al. [11]. (c) United Kingdom: reported cases from Our World in Data [4] and serological prevalence from Public Health England [12–14]. (d) United States: reported cases from Our World in Data [4] and serological prevalence from Walker et al. [15] and Anand et al. [16]. Dashed lines are the simulated cumulative infections, and shaded regions depict the 95% Confidence Interval (CI).

Fig 15 illustrates that our simulations for the early pandemic moments align with national serological research for Brazil, the United Kingdom, and the United States. Notably, Spain is the only country where our simulation deviates significantly.

Hallal et al. [10] conducted two seroprevalence surveys in 133 larger cities across all Brazilian states, utilizing random household and individual selection while excluding children under one year. The first survey, conducted from May 14–21, 2020, included 25,025 individuals, estimating a seroprevalence of 1.9% (95% CI: 1.7–2.1), and the second survey, conducted from June 4–7, 2020, included 31,165 individuals, estimating a seroprevalence of 3.1% (95% CI: 2.8–3.4). In comparison, our model estimated cumulative infections for Brazil as 2.36% (95% CI: 2.17–2.56) on May 14, 2020, and 4.06% (95%CI: 3.72–4.44) on June 04, 2020.

Perez-Gómez et al. [11] conducted a representative cohort study of the noninstitutionalized Spanish population, with 68,287 participants between April 27, 2020, and June 22, 2020. They estimated a seroprevalence in Spain of 6% (95% CI: 5.7–6.4) for this period. In contrast, our model estimated a prevalence of 15.5% (95% CI: 12.4–18.8) on April 27, 2020.

Public Health England conducted four serological surveys in the first wave in England [12–14], which serve as a reference for our simulations in the United Kingdom. The first two surveys [12] were based on healthy blood donors aged 17–69 years, aligning well with our simulations. However, the last two surveys [13, 14] included healthy blood donors aged 17 years and older, reducing seroprevalence and causing our model to estimate the prevalence higher than these surveys. Public Health England attributes this decrease in prevalence to demographic variations in the donor pool, such as the inclusion of donors aged 70 years and older, who were previously restricted during lockdown, and also considers waning antibodies as a potential contributing factor [13, 14].

Our prevalence simulations for the United States align with two serological surveys based on dialysis patients. Walker et al. [15] estimated a prevalence of 5.8% (95% CI: 5.4–6.2) among 12,932 dialysis patients from May 27, 2020, to July 1, 2020. Additionally, Anand et al. [16] estimated a prevalence of 9.3% (95% CI: 8.8–9.9) based on a sample of 28,503 randomly selected adult patients receiving dialysis during July 2020.

### 4.4 Forecasting analysis

**Fig 16:**
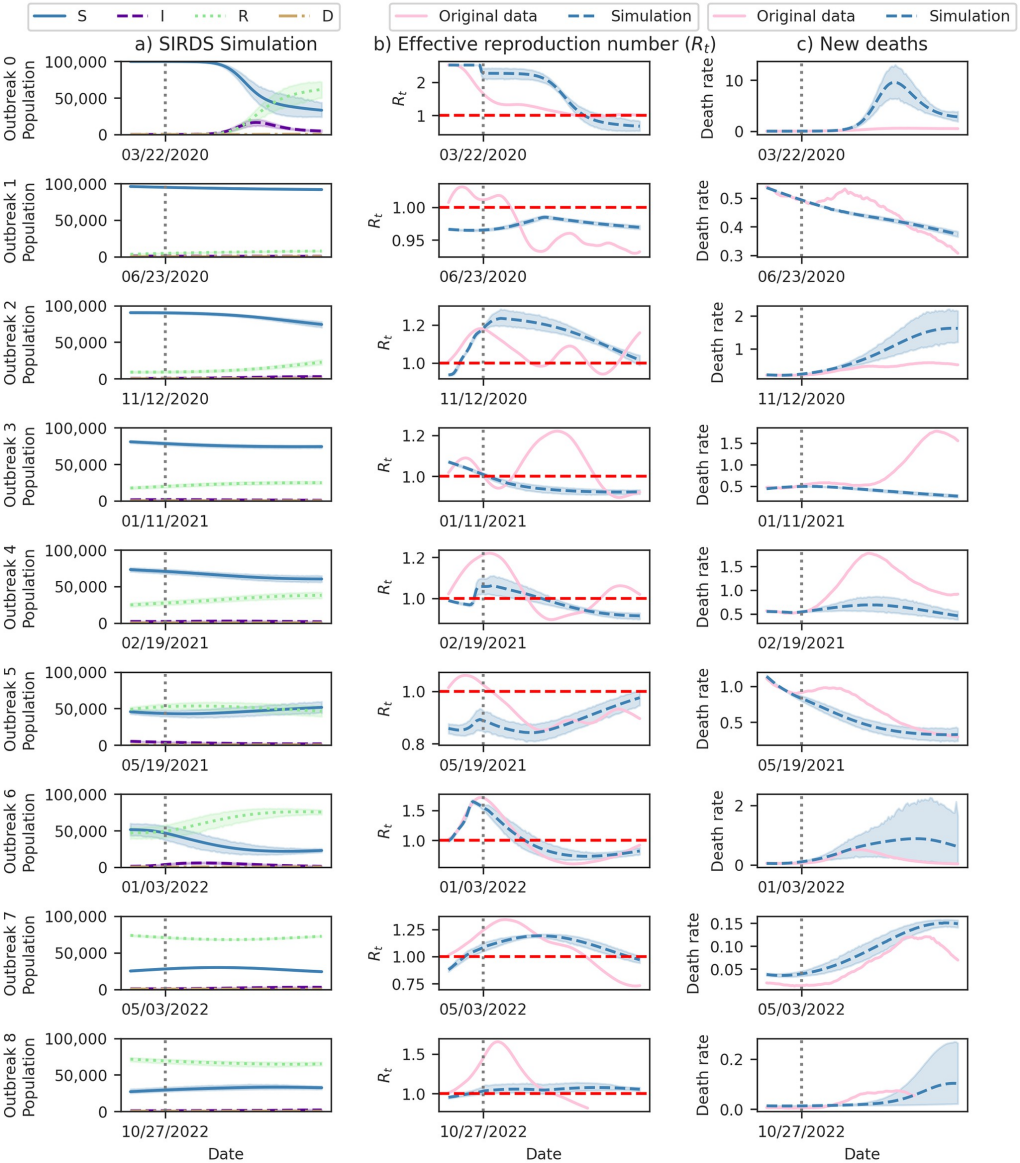
Forecasting Covid-19 dynamics in Brazil for different outbreaks. Each row represents a distinct outbreak. Column (a) displays SIRDS simulation outputs for Susceptible (S), Infected (I), Recovered (R), and Deceased (D) compartments. Column (b) compares the effective reproduction number (*R*_*t*_) from Section 3.2 with model simulations, including a dashed line at the reference value (*R*_*t*_ = 1). Column (c) contrasts the rate of new deaths per 100,000 inhabitants in a 7-day moving average from the Mortality Information System [3] with new deaths estimated by model simulations. Vertical dotted lines mark the 21^*st*^ day inside the outbreak, the maximum fit date for forecasting the next 90 days. Shaded regions indicate the 95% Confidence Interval (CI).

Fig 16 provides a comprehensive summary of the three-month ahead forecasts generated by our model for the nine observed Covid-19 outbreaks in Brazil. While Table 4 indicates a generally good fit for the outbreaks, it also highlights an increasing error trend for predictions over more extended periods. The initial outbreak (Outbreak 0) had the highest error, suggesting insufficient data for accurate prediction. Accurately estimating new outbreaks during the forecasting horizon, as observed in Outbreaks 2, 3, and 4, poses a significant challenge.

**Table 4:** Fit and prediction errors for the Covid-19 outbreaks in Brazil.

Outbreak	Fitting period error	Forecasting period errors^a^		
		1^*st*^ month	2^*nd*^ month	3^*rd*^ month
0	0.40 (0.39–0.41)	2.22 (1.80–2.60)	13.17 (9.10–17.35)	8.20 (6.23–10.41)
1	0.06 (0.06–0.06)	0.11 (0.10–0.11)	0.13 (0.12–0.14)	0.13 (0.11–0.15)
2	0.06 (0.06–0.06)	0.41 (0.27–0.56)	1.07 (0.60–1.69)	1.83 (1.04–2.82)
3	0.07 (0.07–0.07)	0.22 (0.20–0.24)	0.63 (0.59–0.69)	0.84 (0.82–0.87)
4	0.08 (0.08–0.08)	0.46 (0.40–0.53)	0.66 (0.59–0.72)	0.60 (0.52–0.68)
5	0.10 (0.10–0.10)	0.39 (0.31–0.48)	0.46 (0.36–0.57)	0.58 (0.46–0.70)
6	0.12 (0.11–0.13)	1.24 (0.89–1.70)	2.48 (1.32–4.06)	10.81 (1.89–27.0)
7	0.15 (0.14–0.15)	1.45 (1.04–1.99)	0.51 (0.37–0.66)	0.70 (0.64–0.75)
8	0.16 (0.16–0.17)	0.83 (0.76–0.87)	0.94 (0.81–1.16)	N/A^b^

^*a*^The forecast horizon is presented split into three different months.

^*b*^No actual data is available to calculate the error.

Note: values are presented as the mean with 95% Confidence Interval bounds in parenthesis.

Error: the objective function error, as defined by Eq (12).

Table 5 focuses on presenting the death errors. Notably, in the first month, our model exhibits a maximum PAE of 35% for six of the nine outbreaks in Brazil. However, in the second and third months, our model presents a maximum PAE of 50% for only four outbreaks. This reduction in performance highlights the challenge of forecasting epidemics for long-term periods.

**Table 5:** Percentual absolute error of Covid-19 deaths forecasting outbreaks in Brazil.

Outbreak	Percentual Absolute Error (PAE)^a^		
	1^*st*^ month	2^*nd*^ month	3^*rd*^ month
0	136 (113–159)	1,275 (834–1,691)	777 (584–1,017)
1	7 (6–7)	9 (8–10)	9 (7–11)
2	28 (18–40)	92 (50–153)	172 (93–269)
3	14 (12–16)	41 (38–45)	81 (79–83)
4	35 (30–40)	60 (53–66)	49 (40–58)
5	29 (23–35)	37 (26–47)	49 (39–60)
6	97 (62–142)	214 (109–365)	1,017 (142–2,501)
7	137 (92–189)	44 (30–62)	37 (30–44)
8	19 (14–24)	80 (71–94)	705 (90–1,921)

^*a*^The forecast horizon is presented split into three different months.

Note: values are presented as the mean with 95% Confidence Interval bounds in parenthesis.

**Fig 17:**
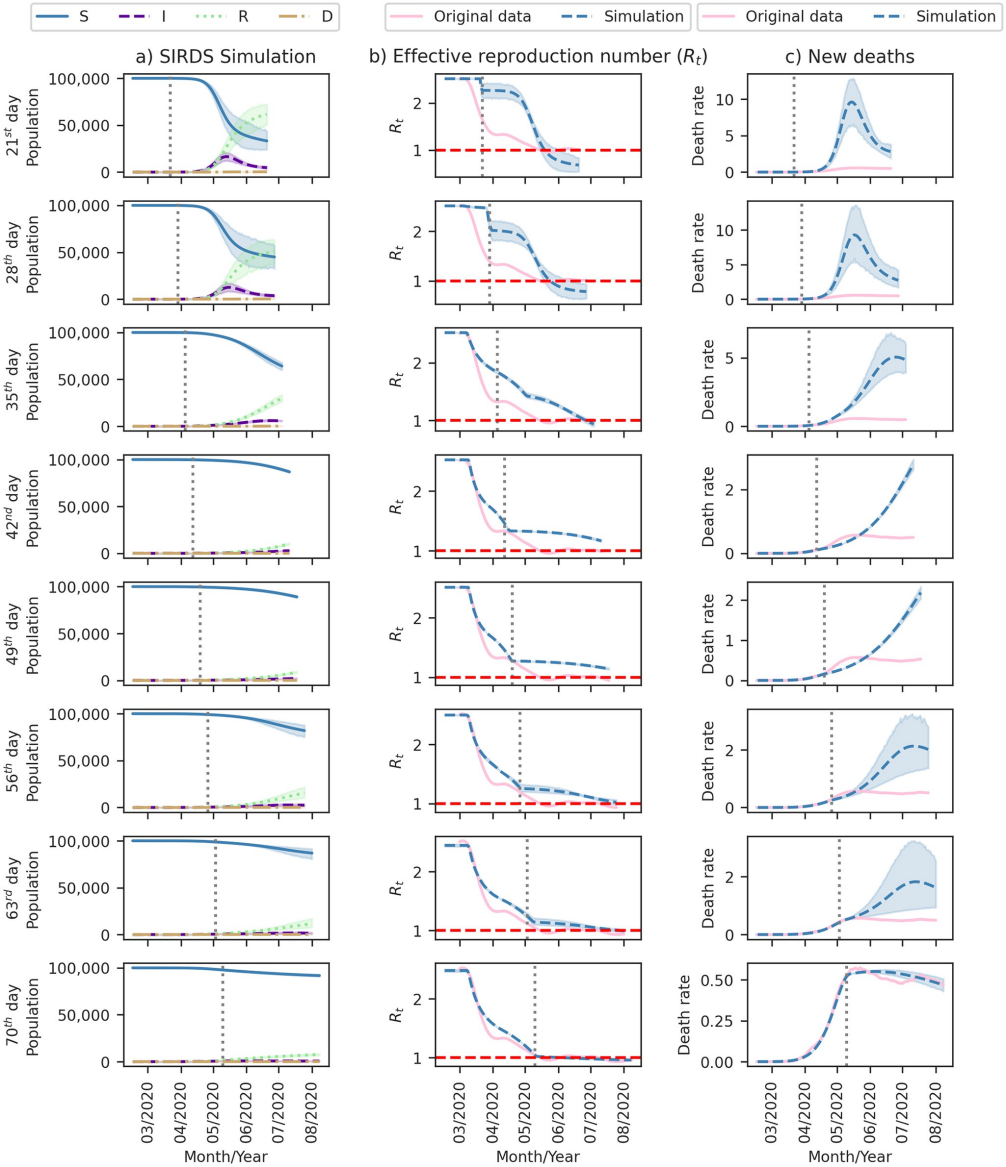
Forecasting Covid-19 dynamics during the initial outbreak in Brazil. Each line represents a day period within the first outbreak to mark the maximum adjustment date for forecasting the next 90 days, and the vertical dotted lines also highlight this date. Column (a) displays SIRDS simulation outputs for Susceptible (S), Infected (I), Recovered (R), and Deceased (D) compartments. Column (b) compares the effective reproduction number (*R*_*t*_) from Section 3.2 with model simulations, including a dashed line at the reference value (*R*_*t*_ = 1). Column (c) contrasts the rate of new deaths per 100,000 inhabitants in a 7-day moving average from the Mortality Information System [3] with new deaths estimated by model simulations. Shaded regions indicate the 95% Confidence Interval (CI).

Fig 17 illustrates that the predictive accuracy of our model improves when the fitting period encompasses more days within the outbreak. By fitting the initial outbreak until its 70^*th*^ day, we achieved reduced death PAE, measuring 1.9% (95% CI: 1.2–2.6), 10.3% (95% CI: 7.4–13.3), and 12.7% (95% CI: 9.7–15.6) for the first, second, and third months, respectively.

### 4.5 Sensitivity analysis

**Fig 18:**
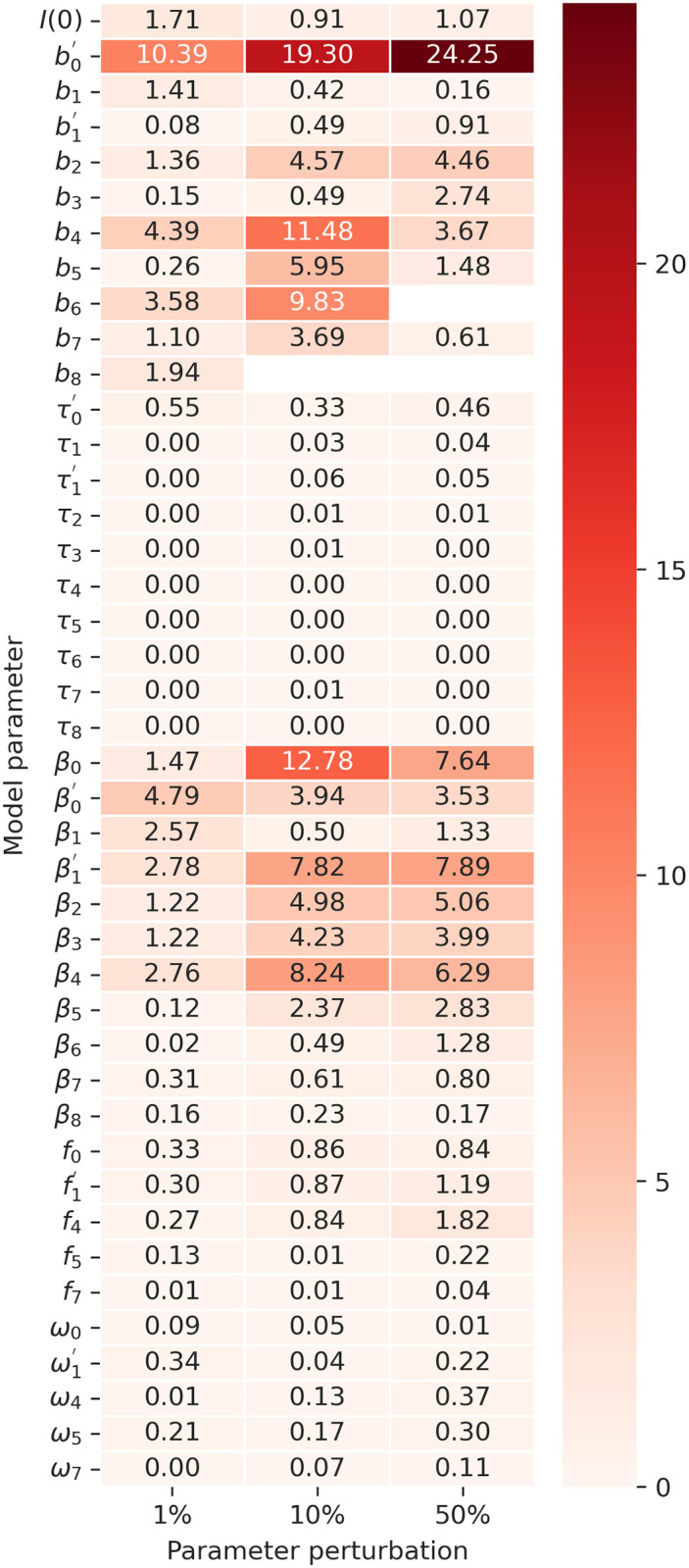
Sensitivity analysis heatmap for perturbations of 1%, 10%, and 50% in optimized parameters with Covid-19 data in Brazil. Each row corresponds to a specific parameter *θ*_*k*_, where *k* denotes the parameter associated with a particular Covid-19 outbreak. When $\theta_{k}^{\prime }$ is mentioned, it represents an adjustment parameter for atypical outbreak *k*. The numerical values in each cell represent the elasticity measured for *θ* under a specific perturbation. The parameters include the initial quantity of infected population *I*(0), the breakpoint indicating the start of an outbreak (*b*), transition days between epidemic periods for fast transitions (*τ*), contact rate (*β*), infection fatality rate probability (*f*), and immunity loss rate (*ω*). Empty cells indicate simulations with errors due to invalid parameter values.

In Fig 18, the impact of perturbations in the first breakpoint parameter (*b*_0_) on the Covid-19 simulation for Brazil is evident, showcasing its significant sensitivity. Generally, breakpoint parameters demonstrate considerable sensitivity. Additionally, parameters related to transmissibility, denoted by *β*, exhibit sensitivity, with the last three showing relatively lower impact. Notably, the initial population infected (*I*(0)) is another parameter demonstrating sensitivity among the model variables.

Conversely, our model exhibits low sensitivity in two crucial epidemiological parameters, namely lethality (*f*) and loss of immunity (*ω*). About lethality, we observed some sensitivity for $f_{1}^{\prime }$ and *f*_4_ when perturbed by 50%. An additional parameter introduced in our model to facilitate smooth transitions between epidemic periods (*τ*) demonstrated low sensitivity, with $\tau_{0}^{\prime }$ being the only instance where we observed some elasticity.

## 5 Discussion

In this study, we developed a novel mathematical epidemiological model with time-varying parameters driven by fuzzy transitions between epidemic periods. The formulation of this model originated from the considerations outlined in Section 3.4.2. We applied and validated our model using Covid-19 data from Brazil, Spain, the United Kingdom, and the United States, demonstrating its robustness and generalization capabilities (Section 4.2). Comparative analyses with seroprevalence research in Section 4.3 illustrated good model fit, aligning well with the available evidence. Furthermore, our model exhibited utility in forecasting Covid-19 outbreaks, as presented in Section 4.4. The sensitivity analysis in Section 4.5 emphasized the crucial role of the time-varying property in capturing the dynamics of the Covid-19 pandemic over three years, particularly concerning changes in transmissibility and breakpoints between epidemic periods. We use this model to present a retrospective analysis of the pandemic in Brazil in Section 4.1.

While several studies have leveraged fuzzy theory for Covid-19 pandemic modeling to account for complexity and uncertainties [64–68], our approach differs from others to employ fuzzy theory in the transitions between epidemic periods, turning the modeling of epidemic period transitions more aligned with our observations of the Covid-19 pandemic. This feature provides a comprehensive view of the epidemiological parameter dynamics, as evidenced in Fig 14, which depicts the time-varying trends for *R*_0_, IFR, and the protection period in the Brazilian context.

We used the proposed model in this work to reproduce the first three Covid-19 pandemic years in Brazil with great reasonability. The estimated *R*_0_ of 2.44 (95% CI: 2.42–2.46) aligns with other works [27, 48]. Notably, the time-varying *R*_0_ during the first wave is in line with the adjusted *R*_0_ of 0.91 (95% CI: 0.83–1.01) identified by Nouvellet et al. [27], reflecting the initial reduction in human mobility.

Marra and Quartin [17], using the same database of Hallal et al. [10], estimated the IFR for Brazil as 1.03% (95% CI: 0.88%–1.22%) for serological tests collected between May and June 2020, while our model estimated an IFR of 0.62% (95% CI: 0.57–0.68) for the period until June 24, 2020. Our estimate is consistent with other studies that investigated the IFR in the first wave, such as the study conducted by Picon et al. [18], which estimated an IFR of 0.60% (95% CI, 0.49%–0.74%) considering antibody prevalence in a Brazilian city, Silveira et al. [19], which estimated an IFR of 0.38% (95% CI: 0.21%–0.80%) considering antibody prevalence in a Brazilian state, and Verity et al. [60], which estimated an IFR of 0.66% (95% CI: 0.39%–1.33%) using early PCR tests in China.

The duration of protection against Covid-19 is still uncertain, as research suggests a fast decay of coronavirus antibodies [10]. While some studies indicate that antibody protection could last between five and seven months [69], others suggest that T and B cells may extend protection [70]. Furthermore, there is a risk of reinfection within 90 days of the previous infection [61, 71], and also the emergence of new variants that may escape the protection given by a previous infection or vaccine [7, 61].

Ferrante et al. [72] proposed an epidemic model that estimated a protection period of 240 days for Covid-19 based on data from the first and second waves in Manaus/AM. Similar to our model, that estimated a protection period of 234 days (95% CI: 206–262) for the early pandemic moments. Morris et al. [71] used SARS-CoV-2 genomic surveillance data from Johns Hopkins and found a median interval of 377 days between the first infection and reinfection. However, another empirical study showed that reinfection peaks with intervals of six months in South Africa [61].

The protection period is, without a doubt, the epidemiological parameter of Covid-19 with the most significant uncertainty, as can be seen in our simulation with data from Brazil in Fig 14 and for the other countries in S5 Appendix. Our sensitivity analysis reinforces this uncertainty, which shows that the protection period is a parameter with low sensitivity.

Estimating the underreporting of cases is a critical factor in comprehending the actual magnitude of an epidemic. In the early stages of the Covid-19 pandemic in Brazil, several studies focused on addressing this issue. For instance, Reis et al. [73] utilized a mathematical model and estimated that only 10% of Covid-19 infections in Brazil were reported until April 6, 2020. Our model suggests an even more significant underreporting, indicating that Brazilian health authorities reported approximately 4.3% (95% CI: 4.0–4.6) of infections during the same period. Bastos et al. [74], also employing a mathematical model, estimated a range of 8–16 times more infections than reported cases until May 31, 2020, which aligns with our model that estimated a factor of 15 (95% CI: 13–16) for the same period. Our findings are consistent with a machine learning model proposed by Noh and Danuser [75], which estimated that around 20% of infections were reported in Brazil until September 3, 2020.

We recognize that serological surveys are considered the most reliable method for assessing the underreporting of Covid-19 cases. While the comprehensive Covid-19 serological survey in Brazil conducted by Hallal et al. [10] did not explicitly calculate the underreporting ratio, we estimated an underreporting factor of 9.1 (95% CI: 8.2–10.0), based on their prevalence study on June 4–7, 2020. Our model approximated the factor derived from Hallal et al. [10], estimating a factor of 12.6 (95% CI: 11.6–13.9) until June 7, 2020.

It is important to note that serological surveys also have limitations as they cannot differentiate between historical and current infections or distinguish antibodies resulting from natural exposure and vaccination [20]. Conducting a serological survey after around three years (1,050 days) becomes increasingly challenging, and we have not found recent studies employing this method in the Brazilian context. Therefore, epidemiological modeling is crucial in analyzing a prolonged epidemic such as Covid-19 in Brazil. Our proposed model competes well with solid epidemiological evidence noted in the first wave and successfully captures the mortality rate trends and *R*_*t*_ in other outbreaks. To our knowledge, our model is the first comprehensive investigation of the first three years of Covid-19 in Brazil.

Still, regarding the underreporting of Covid-19 cases in Brazil, our model suggests an increase in underreporting factors after the first pandemic year. We conjecture that the model caught an under-ascertainment phenomenon, where infected individuals do not seek healthcare [20]. Several factors likely contributed to this population behavior, including a sense of reduced risk among the population due to vaccination [1, 76], the predominance of mild Omicron infections [7], and the availability of self-tests [77].

The official data about Brazil revealed a 79% reduction in CFR during the 2022 year compared to the first pandemic year. Our model estimated an even more substantial 94% (95% CI: 93–95) reduction in IFR for the same period, likely associated with increased underreporting during the Omicron phase, as previously discussed. Likewise, for the Omicron phase, other mathematical models by Xavier et al. [28] estimated a 41% reduction in IFR for Brazil, Liu et al. [21] reported a 78.7% reduction (95% CI: 66.9%–85.0%) in South Africa, and Yan and Shaman [22] observed IFR reductions in nine South African provinces.

### 5.1 Limitations

While our work effectively captures the dynamic changes in epidemiological parameters across outbreaks, we must recognize certain limitations inherent in our modeling approach.

Firstly, we maintained a fixed recovery period of 8 days (*γ* = 1/8), as discussed in Section 3.5.1. Our empirical observation drove this decision that introducing time variability to *γ* significantly increased computational costs for parameter optimization without yielding substantial improvements in model outcomes.

Moreover, our model does not explicitly include an *Exposed* (E) compartment, despite the common suggestion in the literature to incorporate an incubation period for modeling Covid-19 [78–80]. We addressed this limitation by assuming that our compartmentalization of the *Infected* (I) compartment implicitly considers aspects of asymptomatic and pre-symptomatic infections. Our empirical observation indicates that the model aligns well with the observed series of deaths and *R*_*t*_ even with this simplification.

It is crucial to note that we relied on notification dates reported by health authorities rather than the actual event dates for data from Spain, the United Kingdom, and the United States. This divergence in data sources introduces uncertainties and may impact the precision of our generalization analyses.

## 6 Conclusion

We conclude that Brazil effectively controlled the initial wave of Covid-19 by reducing the *R*_*t*_. However, during the second wave, the country encountered challenges in rapidly reducing *R*_*t*_ as it had in the first wave, resulting in the highest proportion of Covid-19 deaths occurring during this period. Our model estimates that, during the second wave, the recovered population surpassed the susceptible population for the first time, indicating significant population exposure to the virus across in this wave. The combination of it with a 77% vaccination rate against Covid-19 [1] and evidence of the Omicron variant being less lethal [7], likely contributed to the substantial reduction in the IFR observed in 2022, despite the high number of infections.

To summarize, public health measures such as reducing human mobility and mass vaccination have been effective against Covid-19 outbreaks, as evidenced by the data in Brazil. We observed a significant reduction in human mobility in the early moment, and in 2022, the country already had a significant proportion of the population vaccinated. However, adequate public health measures were absent in the second wave, leading to elevated mortality rates.

Furthermore, this study introduces a novel mathematical epidemiological model with time-varying parameters driven by fuzzy transitions between epidemic periods, demonstrating its ability to capture the pandemic dynamics over three years. The methodology and insights presented here can inform policymakers in shaping effective strategies to combat future epidemic outbreaks.

Our future research efforts include extending the model application to estimate epidemic parameters individually at the municipal level, enabling more accurate quantification of intervention effectiveness, such as social isolation and vaccination. Additionally, we aim to conduct a deeper forecasting analysis using our model by leveraging larger datasets with more location samples and conducting benchmark comparisons with other machine-learning models across multiple Covid-19 outbreaks.

## Supporting information

S1 Appendix Epidemiological time series of Covid-19 for Spain, the United Kingdom, and the United States. (ODT)

S2 Appendix Effective reproduction number (*R*_*t*_) for Spain, the United Kingdom, and the United States. (ODT)

S3 Appendix Time series of Covid-19 Case Fatality Rate (CFR) for Spain, the United Kingdom, and the United States. (ODT)

S4 Appendix Fuzzy variables fitted for smoothing transitions between epidemic periods. (ODT)

S5 Appendix Time-varying model parameters fitted for Covid-19 in Spain, United Kingdom, and United States. (ODT)

S6 Appendix Comprehensive analysis of simulation results for Covid-19 in Spain, United Kingdom, and United States. (ODT)
