# Supplementary material for: Estimating time-varying epidemiological parameters and underreporting of Covid-19 cases in Brazil using a mathematical model with fuzzy transitions between epidemic periods
Source: PLoS One. 2024 Jun 17;19(6):e0305522. doi: 10.1371/journal.pone.0305522 (PMC11182538; doi:10.1371/journal.pone.0305522)
Supplement: Supplementary file 1 [file pone.0305522.s001.odt]

S1 Appendix. Epidemiological Time Series of Covid-19 for Spain, the United Kingdom, and the United States.

S1A. Time series of Covid-19 in Spain at the reported date on the National Monitoring Panel.(a) new reported cases and (b) new reported deaths. Data from Our World in Data [1].

S1B. Time series of Covid-19 in United Kingdom at the reported date on the National Monitoring Panel. (a) new reported cases and (b) new reported deaths. Data from Our World in Data [1].

S1C. Time series of Covid-19 in United States at the reported date on the National Monitoring Panel.(a) new reported cases and (b) new reported deaths. Data from Our World in Data [1].

References

1. Ritchie H, Mathieu E, Rodés-Guirao L, Appel C, Giattino C, Ortiz-Ospina E, et al.. Coronavirus Pandemic (COVID-19); 2020 [cited 2023 July 14]. In: Our World in Data [Internet]. Available from: https://ourworldindata.org/coronavirus.
