# Supplementary material for: Estimating time-varying epidemiological parameters and underreporting of Covid-19 cases in Brazil using a mathematical model with fuzzy transitions between epidemic periods
Source: PLoS One. 2024 Jun 17;19(6):e0305522. doi: 10.1371/journal.pone.0305522 (PMC11182538; doi:10.1371/journal.pone.0305522)
Supplement: Supplementary file 2 [file pone.0305522.s002.odt]

S2 Appendix. Effective reproduction number (Rt) for Spain, the United Kingdom, and the United States.

S2A. Effective reproduction number (Rt) for Covid-19 in Spain. The dashed horizontal line represents the reference value (Rt = 1) used to monitor epidemics. The Rt time series alternates between outbreak periods (solid line) and non-outbreak periods (dotted line). Nine outbreak periods, labeled from #0 to #9, were identified.

S2B. Effective reproduction number (Rt) for Covid-19 in United Kingdom. The dashed horizontal line represents the reference value (Rt = 1) used to monitor epidemics. The Rt time series alternates between outbreak periods (solid line) and non-outbreak periods (dotted line). Nine outbreak periods, labeled from #0 to #9, were identified.

S2C. Effective reproduction number (Rt) for Covid-19 in United States. The dashed horizontal line represents the reference value (Rt = 1) used to monitor epidemics. The Rt time series alternates between outbreak periods (solid line) and non-outbreak periods (dotted line). Nine outbreak periods, labeled from #0 to #10, were identified.
