# Supplementary material for: Estimating time-varying epidemiological parameters and underreporting of Covid-19 cases in Brazil using a mathematical model with fuzzy transitions between epidemic periods
Source: PLoS One. 2024 Jun 17;19(6):e0305522. doi: 10.1371/journal.pone.0305522 (PMC11182538; doi:10.1371/journal.pone.0305522)
Supplement: Supplementary file 4 [file pone.0305522.s004.odt]

S4 Appendix: Fuzzy variables fitted for smoothing transitions between epidemic periods.

S4A. Fuzzy Variables Fitted for Smoothing Transitions Between Epidemic Periods in Brazil. (a) Fuzzy Variable with Partitions Representing Epidemic Periods with Fast Transitions. (b) Fuzzy Variable with Partitions Representing Epidemic Periods with Slow Transitions. The colors represent partitions, and shaded regions depict the 95\% Confidence Interval (CI).

S4B. Fuzzy Variables Fitted for Smoothing Transitions Between Epidemic Periods in Spain. (a) Fuzzy Variable with Partitions Representing Epidemic Periods with Fast Transitions. (b) Fuzzy Variable with Partitions Representing Epidemic Periods with Slow Transitions. The colors represent partitions, and shaded regions depict the 95\% Confidence Interval (CI).

S4C. Fuzzy Variables Fitted for Smoothing Transitions Between Epidemic Periods in United Kingdom. (a) Fuzzy Variable with Partitions Representing Epidemic Periods with Fast Transitions. (b) Fuzzy Variable with Partitions Representing Epidemic Periods with Slow Transitions. The colors represent partitions, and shaded regions depict the 95\% Confidence Interval (CI).

S4D. Fuzzy Variables Fitted for Smoothing Transitions Between Epidemic Periods in United States. (a) Fuzzy Variable with Partitions Representing Epidemic Periods with Fast Transitions. (b) Fuzzy Variable with Partitions Representing Epidemic Periods with Slow Transitions. The colors represent partitions, and shaded regions depict the 95\% Confidence Interval (CI).
