# Supplementary material for: Estimating time-varying epidemiological parameters and underreporting of Covid-19 cases in Brazil using a mathematical model with fuzzy transitions between epidemic periods
Source: PLoS One. 2024 Jun 17;19(6):e0305522. doi: 10.1371/journal.pone.0305522 (PMC11182538; doi:10.1371/journal.pone.0305522)
Supplement: Supplementary file 5 [file pone.0305522.s005.odt]

S5 Appendix: Time-varying model parameters fitted for Covid-19 in Spain, United Kingdom, and United States.

S5A. Time-varying model parameters fitted for Covid-19 in Spain. (a) Basic reproduction number (R0) varying with time (t). (b) Infection Fatality Rate (IFR) varying with t. (c) Days to loss of immunity (Ω) varying with t. Shaded regions depict the 95% Confidence Interval (CI).

S5B. Time-varying model parameters fitted for Covid-19 in United Kingdom. (a) Basic reproduction number (R0) varying with time (t). (b) Infection Fatality Rate (IFR) varying with t. (c) Days to loss of immunity (Ω) varying with t. Shaded regions depict the 95% Confidence Interval (CI).

S5C. Time-varying model parameters fitted for Covid-19 in United States. (a) Basic reproduction number (R0) varying with time (t). (b) Infection Fatality Rate (IFR) varying with t. (c) Days to loss of immunity (Ω) varying with t. Shaded regions depict the 95% Confidence Interval (CI).
