# Supplementary material for: Estimating time-varying epidemiological parameters and underreporting of Covid-19 cases in Brazil using a mathematical model with fuzzy transitions between epidemic periods
Source: PLoS One. 2024 Jun 17;19(6):e0305522. doi: 10.1371/journal.pone.0305522 (PMC11182538; doi:10.1371/journal.pone.0305522)
Supplement: Supplementary file 6 [file pone.0305522.s006.odt]

S6 Appendix: Comprehensive analysis of simulation results for Covid-19 in Spain, United Kingdom, and United States.

S6A. Comprehensive analysis of simulation results for Covid-19 in Spain. (a) Model outcomes for an eight-day recovery period detailing the population compartments: Susceptible, Infected, Recovered, and Deceased. (b) Time series comparison between the effective reproduction number (Rt) estimated directly from reported Severe Acute Respiratory Syndrome (SARS) cases and Rt calculated by model simulations. (c) Time series comparison between new cases reported by health authorities and new infections in model simulations. (d) Time series comparison between new deaths reported by health authorities and new deaths in model simulations. (e) Time series comparison between cumulative cases reported by health authorities and cumulative infections in model simulations. (f) Time series comparison between cumulative deaths reported by health authorities and cumulative deaths in model simulations. Shaded regions depict the 95% Confidence Interval (CI).

S6B. Comprehensive analysis of simulation results for Covid-19 in United Kingdom. (a) Model outcomes for an eight-day recovery period detailing the population compartments: Susceptible, Infected, Recovered, and Deceased. (b) Time series comparison between the effective reproduction number (Rt) estimated directly from reported Severe Acute Respiratory Syndrome (SARS) cases and Rt calculated by model simulations. (c) Time series comparison between new cases reported by health authorities and new infections in model simulations. (d) Time series comparison between new deaths reported by health authorities and new deaths in model simulations. (e) Time series comparison between cumulative cases reported by health authorities and cumulative infections in model simulations. (f) Time series comparison between cumulative deaths reported by health authorities and cumulative deaths in model simulations. Shaded regions depict the 95% Confidence Interval (CI).

S6C. Comprehensive analysis of simulation results for Covid-19 in United States. (a) Model outcomes for an eight-day recovery period detailing the population compartments: Susceptible, Infected, Recovered, and Deceased. (b) Time series comparison between the effective reproduction number (Rt) estimated directly from reported Severe Acute Respiratory Syndrome (SARS) cases and Rt calculated by model simulations. (c) Time series comparison between new cases reported by health authorities and new infections in model simulations. (d) Time series comparison between new deaths reported by health authorities and new deaths in model simulations. (e) Time series comparison between cumulative cases reported by health authorities and cumulative infections in model simulations. (f) Time series comparison between cumulative deaths reported by health authorities and cumulative deaths in model simulations. Shaded regions depict the 95% Confidence Interval (CI).
